# The GPI-Anchored Aspartyl Proteases Encoded by the *YPS1* and *YPS7* Genes of *Candidozyma auris* and Their Role Under Stress Conditions

**DOI:** 10.3390/jof11080573

**Published:** 2025-08-01

**Authors:** Alvaro Vidal-Montiel, Daniel Clark-Flores, Eulogio Valentín-Gómez, Juan Pedro Luna-Arias, Erika Rosales-Cruz, César Hernández-Rodríguez, Lourdes Villa-Tanaca, Margarita Juárez-Montiel

**Affiliations:** 1 Laboratorio de Biología Molecular de Bacterias y Levaduras, Departamento de Microbiología, Escuela Nacional de Ciencias Biológicas, Instituto Politécnico Nacional, Prol. de Carpio y Plan de Ayala. Col. Sto. Tomás, Mexico City 11340, Mexico; alvaro301013@gmail.com (A.V.-M.); clark3dd@gmail.com (D.C.-F.); chdez@ipn.mx (C.H.-R.); 2 Departamento de Microbiología y Ecología, Universidad de Valencia, 46100 Burjassot, Valencia, Spain; eulogio.valentin@uv.es; 3 Grupo de Investigación Infección Grave, Instituto de Investigación La Fe, 46026 Valencia, Spain; 4 Departamento de Biología Celular, Centro de Investigación y de Estudios Avanzados del Instituto Politécnico Nacional (Cinvestav-IPN), Mexico City 07360, Mexico; jpluna@cinvestav.mx; 5 Laboratorio de Investigación en Hematopatología, Departamento de Morfología, Escuela Nacional de Ciencias Biológicas, Mexico City 11340, Mexico; erika_encb@hotmail.com

**Keywords:** *Candidozyma auris*, yapsin, aspartyl protease, glycosylphosphatidylinositol GPI, cell wall integrity, stress conditions, transcriptome

## Abstract

*Candidozyma auris* is a multidrug-resistant, thermo- and osmotolerant yeast capable of persisting on biotic and abiotic surfaces, attributes likely linked to its cell wall composition. Here, seven putative genes encoding yapsins, aspartyl proteases GPI-anchored to the membrane or cell wall, were identified in the genomes of *C. auris* CJ97 and 20-1498, from clades III and IV, respectively. The *C. auris YPS1* gene is orthologous to the *SAP9* of *C. albicans*. The *YPS7* gene is orthologous to *YPS7* in *C. glabrata* and *S. cerevisiae*, so that they may share similar roles. An in silico analysis suggested an interaction between pepstatin and the catalytic domain of Yps1 and Yps7. Although this inhibitor, when combined with caffeine, had a subtle effect on the growth of *C. auris*, it induced alterations in the cell wall. *CauYPS1* and *CauYPS7* expression increased under nutrient starvation and NaCl, and at 42 °C. The transcriptome of the 20-1498 strain suggests that autophagy may play a role in thermal stress, probably degrading deleterious proteins or maintaining cell wall and vacuolar homeostasis. Therefore, CauYps1 and CauYps7 may play a role in the cell wall integrity of *C. auris* in stress conditions, and they could be a target of new antifungal or antivirulence agents.

## 1. Introduction

Aspartyl proteases are enzymes that have gained attention due to their involvement in the infection processes of pathogenic fungi. Yeast aspartyl proteases, or yapsins, are anchored to the cell wall through covalent bonds to β-1,6-glucans or the cell membrane via the GPI (glycosylphosphatidylinositol) group. They participate in cell wall remodeling by processing proteins located here, a process essential for the integrity of this structure during morphogenesis, and adaptation to environmental changes adhesion, biofilm development, and nutrient acquisition by degrading extracellular proteins, such as host tissue and immune defense proteins [1,2,3,4]. Since 1990, when Egel-Mitani et al. [5] described in *S. cerevisiae* GPI-aspartyl proteases capable of partially complementing kex2 mutants by processing the α-mating factor precursor when it is overexpressed, it has been shown that, in contrast to other aspartyl proteases with an affinity to hydrophobic residues, *S. cerevisiae* yapsins’s cleave dibasic residues [6]. Then, Bourbonnais et al. [7] showed that the Yps1 of *S. cerevisiae* also cleaves monobasic residues, unlike Kex2. Similarly, Sap9 and Sap10 of *C. albicans* partially complemented kex2 mutants by processing monobasic and dibasic residues of peptides. Although Sap9 and Sap10 *C. albicans* still retain the nomenclature of the secreted aspartyl protease (Sap), proteases without GPI modification, in silico, molecular, and biochemical studies have shown that they differ from other Sap members. For example, they exhibit enzymatic activity over a broad range of pH values, even at a neutral (physiological) pH, have their distinct cleavage preferences, and are cell-surface-associated proteases that function in cell wall integrity and the interaction with human epithelial cells and neutrophils [2].

Yapsins are synthesized as zymogens, containing an N-terminal signal peptide removed after translocation into the endoplasmic reticulum by a signal peptidase. These enzymes present a catalytic domain with two aspartic residues, or at least one, in the Asp-Thr(Ser)-Gly(DT(S)G) consensus, which is crucial for their enzymatic function, the N-terminal propeptide removed in the Golgi apparatus at the dibasic or monobasic residues by Kex2 or by yapsin peptidases [1,2,8]. The C-terminal is a hydrophobic domain for transient binding to the ER membrane and the ω site where the protein is linked to the GPI anchor. The ω site is at the consensus sequence [NSGADC]–[GASVIETKDLF]–[GASV]–X(4,19)–[FILMVAGPSTCYWN]. Like other aspartyl proteases from the A1 family, they contain four cysteines forming disulfide bridges [1,9,10]. It has been observed that yapsins, like Saps, of human pathogenic yeasts, occur as multigene families, and the expansion of these families seems to be related to their success as pathogens and in their survival [11].

*Candidozyma auris* (syn. *Candida auris*) is an opportunistic multidrug-resistant pathogen first described in 2009 by Satoh et al. [12]. Since then, it has been identified as being responsible for hospital outbreaks worldwide, causing systemic infections with high mortality. As a result, the World Health Organization issued an epidemiological alert to identify, notify, and manage cases and outbreaks caused by *C. auris* promptly [13]. Additionally, *C. auris* is thermotolerant and halotolerant, which contributes to its ability to colonize various environments. This yeast also forms pseudohyphae and aggregates under specific conditions [14,15,16,17]. Among other characteristics that distinguish *C. auris* from other pathogenic yeasts are its capacity to form multilayered biofilm and cause invasive infections without forming mycelium, and immune evasion, features attributed in part to the yeast cell wall [18,19,20]. *C. auris* isolates have been grouped into six main geographical clades through phylogenetic analyses. The strains of each clade differ by hundreds of thousands of single-nucleotide polymorphisms (SNPs). Genetic differences between clades have been linked to differences in the cell wall and membrane composition [13,21].

In contrast to *C. albicans*, whose genome carries 10 genes encoding Saps, *C. auris* has a multigene family of 14 or 15 genes encoding these proteins [20,22]. Particularly, Sap3 contributes to extracellular proteolytic activity, biofilm formation, and virulence in animal models, suggesting that its inhibition could be a promising therapeutic strategy [22]. Given the importance of secreted aspartyl proteases as virulence factors in *C. auris*, this study addressed the identification of GPI-anchored aspartyl proteases encoding genes of two strains of clades III and IV each and through the use of the aspartyl protease inhibitor, pepstatin A, and the expression analysis of the *C. auris YPS1* and *YPS7* genes, which seem to be the most probable orthologs of *YPS1*, *YPS7,* and *SAP9* of *S. cerevisiae* and other *Candida*, which encode the standout GPI-proteases in cell wall homeostasis [23,24].

Understanding the molecular mechanisms by which *YPS1* and *YPS7* contribute to stress tolerance in *Candida auris* could aid in the development of therapeutic and control strategies against this yeast.

## 2. Materials and Methods

### 2.1. Strains

In this work, *C. auris* CJ97 [25,26] and 20-1498 [27] strains isolated from the blood of hospitalized patients from Spain and Mexico were used, grouped into clades III and IV, respectively. They were stored frozen at −70 °C and routinely grown in YPD (yeast extract 1%, peptone 2%, and dextrose 2%) at 37 °C with shaking at 100 rpm.

### 2.2. In Silico Analysis

The sequences of putative yapsin-coding genes and putative targets of yapsins were identified through BLAST+ 2.17.0+ analysis and hidden Markov models (HMMs) in the previously reported genomes of the two *C. auris* strains CJ97 and 20-1498 (GCA_034640365.1) [28], and *C. auris* B11220 strain from clade II (GCA_003013715.2). Sequences of the *Saccharomyces cerevisiae* yapsin genes (*ScYPS* 1, 2, 3, 6, and 7) from the genome (https://www.yeastgenome.org/, accessed on 12 January 2024) were used as a template. The predicted open reading frame and 1000 bp upstream (considered the promoter sequence) of each selected gene were analyzed using various tools. The SignalP v6.0 (10 February 2024; https://services.healthtech.dtu.dk/services/SignalP-6.0/) [29], ScanProsite (13 February 2024; https://prosite.expasy.org/scanprosite/), and NetGPI v1.1 (15 March 2024; https://services.healthtech.dtu.dk/services/NetGPI-1.1/) [30] were used to find motifs in protein sequences, while, to explain a possible differential expression of the genes, promoters were analyzed in the YEASTRACT server, looking for probable transcription factors binding sites (TFBSs) (27 June 2024; https://saves.mbi.ucla.edu/) [31]. Moreover, the percentage of similarity and identity of protein sequences was stated with MatGat v2.01 [32].

### 2.3. Phylogeny of C. auris Putative Yapsins

First, proteins were aligned with Clustal X [33]. The most conserved regions of GPI-anchored aspartyl peptidases of *S. cerevisiae*, *C. glabrata* (*Nakaseomyces glabratus*), *C. albicans*, and *C. auris* CJ97 were used to construct the phylogeny, with the IQ-TREE software v2.4.0 [34], using the best substitution model (WAG+F+R4). One thousand bootstrap replicates were applied, and the tree was visualized with Figtree v1.4.5. Accession numbers of protein sequence used were as follows: *S. cerevisiae*: ScYps1 (NP_013221.1), ScYps2 (NP_010428.3), ScYps3 (NP_013222.1), ScYps6 (NP_012305.3), and ScYps7 (NP_010636.1); *C. albicans*: CaSap9 (KAL1572689.1), and CaSap10 (XP_717243.1); *C. glabrata* CgYps1 (XP_449529.1), CgYps2 (XP_445750.2), CgYps3 (XP_445764.1), CgYps4 (XP_445765.1), CgYps5 (XP_445766.1), CgYps6 (XP_445767.1), CgYps7 (XP_444870.1), CgYps8 (XP_445768.1), CgYps9 (XP_445769.1), CgYps10 (XP_445770.1), and CgYps11 (XP_445771.1); and *C. auris* B11220: CauYps1 (XP_028889414.2), CauYps2 (XP_028891323.2), CauYps3 (XP_028888407.2), CauYps4 (XP_028892789.1), CauYps5 (XP_028891322.2), CauYps6 (XP_028890862.2), and CauYps7 (XP_028892266.2).

### 2.4. Prediction of the Secondary and Tertiary Structure of Putative Yapsins

The mature forms of putative yapsins CauYps1 (amino acids 52–108 and 199–566) and CauYps7 (amino acids 57–527) were used to predict the secondary and tertiary structure of *C. auris* Yps proteins. Prediction of the secondary and tertiary structure was carried out in ENDscript v3.0 (18 July 2024; https://endscript.ibcp.fr/ESPript/ENDscript/) [35] and the SWISS-MODEL servers, respectively (12 October 2024; https://swissmodel.expasy.org) [36], using the better models selected by the programs, as templates. Once the theoretical tertiary structures were obtained, their quality was analyzed using the SAVES server (15 October 2024; https://saves.mbi.ucla.edu/).

### 2.5. Molecular Docking of C. auris Putative Yps1 and Yps7

The 3D models of predicted mature and CauYps7 from *C. auris*, obtained from the SWISS-MODEL server, were used for molecular docking analysis with the specific and reversible inhibitor of aspartic proteases, pepstatin A, retrieved from the PDB (PRD_000557). This was modified using the Avogadro 2 software to prepare it for interaction [37]. The docking process was performed in ChimeraX [38] with the aid of AutoDock Vina [39].

To test the potential activity of yapsins on a probable molecular target, a molecular docking analysis was conducted using a peptide (MALWMRLLPLLALLALWGPDPAAAFVNQHLCGSHLVEALYLVCGERGFFYTPKTRREAEDLQVGQVELGGGPGAGSLQPLALEGSLQKRGIVEQCCTSICSLYQLENYCN) of the proinsulin hormone (AAW83741.1). The docking was then performed using the H-Dock server (28 October 2024; http://hdock.phys.hust.edu.cn/) [40], which predicts possible molecular interactions between two proteins.

### 2.6. Evaluation of C. auris Growth Under Different Conditions

The *C. auris* strains CJ97 and 20-1498 were pre-cultured for 24 h in YPD medium at 37 °C with shaking at 100 rpm. A 96-well plate was inoculated with 100 µL of yeast at 0.05 of OD_600_ containing YPD medium with different compounds at a final concentration as indicated: 1.5 M NaCl, 12 mM caffeine, 10 mM H_2_O_2_, 0.05% SDS all from Sigma-Aldrich (St. Louis, MO, USA), in the presence and absence of 25 μM pepstatin A (ChemCruz, Dallas, TX, USA). YPD without any compounds was considered the growth control, and YPD without compounds and inoculum was used as the sterility control. Plates were incubated for 20 h, at 37 °C, with shaking, in a Multiskan FC 5111900 (Thermo Fisher Scientific, Waltham, MA, USA) and OD_620_ measured. Three independent replicates were performed, and the standard deviation and significance were determined by two-way ANOVA with SigmaPlot 15.1 software.

### 2.7. Expression of YPS1 and YPS7 Genes by RT-qPCR

Starting from a 48 h pre-culture in a YPD medium, an early stationary phase culture in YPD was obtained by inoculating yeast at an OD_600_ of 0.05 after 15 h of incubation at 37 °C and 100 rpm. These non-proliferating cultures were harvested at 2400 g for 5 min, washed twice, and inoculated into an equal volume of YNB (USBiological, Swampscott, MA, USA) with 2% dextrose (Sigma-Aldrich, St. Louis, MO, USA) as a carbon source and 0.5% (NH4)_2_SO_4_ (Gibco, Waltham, MA, USA) or BSA (Sigma-Aldrich, St. Louis, MO, USA) as a nitrogen source (YNB+C+N); YNB with only carbon (YNB+C-N) or nitrogen (YNB-C+N), and neither carbon nor nitrogen (YNB-C-N), also were tested. The cultures were incubated at 37 °C with shaking at 100 rpm. To evaluate the effect on gene expression at 42 °C and NaCl, cells were transferred to YPD without/with 1.5 M NaCl, incubated at 37 °C, or to YPD alone and incubated at 42 °C. Two independent experiments, each performed in triplicate, were conducted for each condition.

Cells were harvested after 3 and 6 h to extract total RNA using the Schmitt et al. method [41], based on hot phenol extraction. Cells from the desired condition were resuspended in AE buffer (50 mM sodium acetate, 10 mM EDTA) and disrupted with 10% SDS and pH 5 phenol. Samples were placed in a water bath at 65 °C for 5 min and immediately ultra-frozen for 10 min. After centrifugation at 12,879× *g* for 10 min, the aqueous phase was recovered, followed by two washes with chloroform-isoamyl alcohol. Then, absolute and 70% ethanol with DEPC-treated water were added to precipitate and wash RNA. Finally, it was resuspended in nuclease-free water, and its quantity and quality were assessed.

All the RT-qPCR reagents were from Thermo Fisher Scientific (Waltham, MA; USA) SYBR Select Master Mix. cDNA synthesis was performed using 2 μg of DNA-free RNA, treated with DNase I, and the RevertAid reverse transcriptase, along with the oligo (dT)18 primer, according to the manufacturer’s instructions. SYBR Select Master was used according to the supplier’s instructions, using the primers *YPS1* Fw: CTCGAACGAGGAGGACATCG, Rev: TGCTTACAGTCACCGAG; and *YPS7* Fw: CCTTAAAAACTTCAGAGCAGTAGG, Rev GACCACACCAACAAACGCTC. The reaction was subjected to initial denaturation of 95 °C for 5 min, with subsequent forty-five cycles at 95 °C for 30 s, annealing at 59 °C for 30 s, and extension at 72 °C for 1 min 15 s, in a thermal cycler Corbett Research RG-6000 (Corbett Robotic Inc., San Francisco, CA, USA). The data were analyzed with the 2-ΔΔct methodology [42]. The *ACT1* gene (CJI96_0003605) [43] was used as the normalizing gene, and the control conditions were YNB+C+N at 37 °C and YPD at 37 °C. Reactions were performed in triplicate in two biological experiments.

### 2.8. Transcriptomic Analysis of C. auris 20-1498 Under Heat Stress

The *C. auris* strain 20-1498, belonging to clade IV, was cultured in YPD medium until the early stationary phase. Cells were harvested by centrifugation (2400× *g*, 5 min, 4 °C) and washed twice with minimal YNB medium without supplements. They were then incubated for six hours in complete YNB medium (YNB+C+N) at 37 °C and 42 °C.

Total RNA was extracted using the GeneJET RNA Purification Kit (Thermo Scientific; Waltham, MA, USA) following the manufacturer’s instructions. RNA quality was assessed by RNA integrity number (RIN). Library preparation, based on poly-A enrichment, and sequencing on an Illumina NovaSeq X Plus platform were performed by NOVOGENE (Sacramento, CA, USA). Three biological replicates were sequenced per condition.

Raw reads were processed with Trimmomatic v0.39. The quality of filtered reads was evaluated using FastQC v0.12.1. The reference genome of *C. auris* clade IV B11243 (GCA_003014415.1) was downloaded from FungiDB (https://fungidb.org/fungidb/app/, accessed on 12 January 2024). Clean reads were mapped with STAR v2.7.11b, and read counts per gene were obtained using FeatureCounts v2.1.1. Differential expression analysis was performed in R v4.2.2 using DESeq2 v1.44.0. Genes with log2FoldChange ≥ 0.585 or ≤−0.585 and *padj* <0.05 were considered differentially expressed.

Results were processed in Python v3.13.2. Three-dimensional principal component analysis was performed with scikit-learn v1.2.2 and visualized using plotly v5.15.0. The sample distance matrix was generated by calculating Euclidean distances and represented as a heatmap with seaborn v0.12.0. The volcano plot was constructed from the differential expression analysis using matplotlib v3.7.1. Differentially expressed genes were annotated in FungiDB (https://fungidb.org/fungidb/app/, accessed on 12 January 2024) and assigned putative functions by identifying orthologs in CandidaDB (http://www.candidagenome.org, accessed on 12 January 2024) using the pBLAST tool.

Based on functional annotation, Gene Ontology terms associated with biological processes were retrieved, and over-represented terms were visualized in enrichment plots. Finally, differentially expressed orthologous genes were depicted in a lollipop plot using matplotlib v3.7.1.

Processed RNA-Seq data, including gene-level count data and differential expression analyses, are available in the NCBI Gene Expression Omnibus (GEO) under accession number GSE302916. The corresponding raw sequencing reads have been deposited in the NCBI Sequence Read Archive (SRA) under BioProject accession PRJNA1291775.

### 2.9. Effect of Pepstatin A on the Microscopic Morphology of C. auris

Scanning electron microscopy (SEM): Yeast cells were inoculated at 0.05 OD_600_ in YPD medium without or with different concentrations of pepstatin A (12.5, 25, and 50 μM), 12 mM caffeine, or 0.01% methanol, from an inoculum of 24 h in YPD. Cells were treated for 22 h, harvested, and washed with Sörensen’s PBS. They were then fixed overnight at room temperature by adding 2.5% glutaraldehyde. Cells were washed three times with Sörensen’s PBS and sequentially dehydrated with increasing ethanol concentrations, from 50% to 100%. Then, samples were dried for 3 h at 35 °C/1350 psi using a critical point dryer K850 (Quorum Technologies, Lewes, UK) and observed using a scanning electron microscope Quanta FEG 250 (FEI Company, Eindhoven, The Netherlands).

Atomic force microscopy (AFM): Cells treated or not with 50 mM of pepstatin were harvested and washed three times with sterile distilled water. Cells resuspended in an equal volume of water as a medium were placed on a slide, air-dried, and fixed for 1 h with formaldehyde vapors. Afterwards, slides were observed (TT-AFM., Workshop, SC, USA).

## 3. Results

### 3.1. C. auris Presents a Family of YPS Multigene Encoding Putative Yapsins

Genes encoding putative GPI-anchored aspartyl proteases (yapsins) were identified by Blast and HMMs in the genome of three strains of *C. auris*: B11220 (reference genome), CJ97, and 20-1498 from clades II, III, and IV, respectively. We found 15 putative genes encoding aspartyl proteases based on information retrieved from the bioinformatic tools. Seven of these sequences presented the omega site of GPI anchorage (ω). Both CauYps2 and CauYps5 amino acid sequences do not show the typical catalytic aspartic residue of this type of proteases (Figure S1). The putative yapsins ranged in size from 372 to 703 amino acids. *C. auris* yapsins from the three clades showed identities greater than 95.6% and virtually identical motifs, except for CauYps5. CauYps5 from B11220 (clade II) was shorter at the N-terminus than the yapsins from strains CJ97 and 20-1498, and they shared identities of approximately 50% and 60%, respectively (Figure S1).

Additionally, to the two catalytic aspartic residues and the ω site, the N-terminal signal peptide and the typical KR sites, cleavage by Kex2 or autoprocessing at the N-terminal propeptide and the C-terminal, before the ω site, N-glycosylation sites, and low *pI*, all characteristics of the yapsins, were also predicted. The sequences of the *C. auris* CJ97 yapsins were usually more conserved with their cognate sequences in B11220 (Figures S1 and S2); thereby, the sequences of the putative yapsins of the last two strains were used for the in silico analysis.

### 3.2. Yps1 and Yps7 of C. auris are Orthologous to the Yapsins of C. albicans, C. glabrata (Nakaesomyces glabratus), and S. cerevisiae

**Figure 1:**
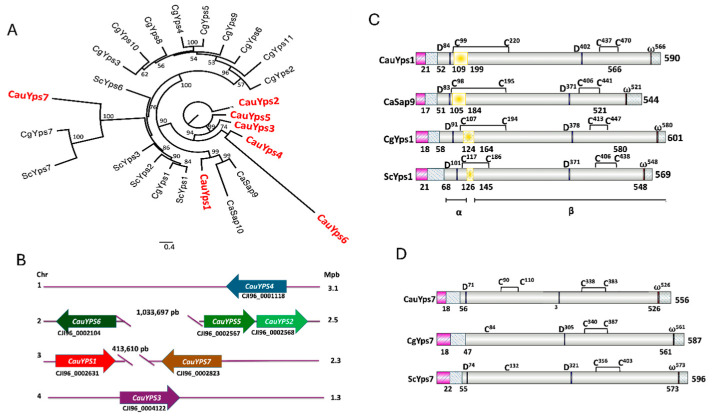
Characteristics of *C. auris* predicted yapsins. (**A**) Phylogenetic analysis of the yapsins multigene family of yeasts. The maximum likelihood reconstruction was performed using amino acid sequences of *S. cerevisiae* (Sc), *C. albicans* (Ca), *C. glabrata* (Cg), and *C. auris* (Cau) in the IQ-TREE software with 1000 bootstrap replicates. Figtree services were used for visualization and edition, and bootstrap support with a confidence level >50% was included in branches. Protein accession numbers were indicated in Section 2. The scale bar shows the number of substitutions per position. (**B**) Schematic representation of *C. auris YPS* gene loci. (**C**) Schematic representation of the domains or motifs of predicted CauYps1 and its homologs of *C. albicans* (Sap9), *C. glabrata* (CgYps1), and *S. cerevisiae* (ScYps1). This yapsin undergoes self-processing in an internal loop (yellow rectangle) and remains split into two subunits (α and β) through a disulfide bridge. The numbers below indicate the numbers where processing takes place. Active aspartic residues (D), omega site (ω), and cysteines of the disulfide bridges (C) are indicated: pink rectangle: signal peptide, grey–blue rectangle: propeptide. (**D**) Motifs of CauYps7 predicted and its homologs yapsin of *C. glabrata* (CgYps7) and *S. cerevisiae* (ScYps7). CgYps7 and ScYps7 did not show one of the cysteines (C) involved in the disulfide bridges, and *CgYPS7* did not show the first catalytic aspartic residue (D).

To examine the phylogenetic relationship between the putative yapsins of *C. auris* and those of other yeast species, an alignment of the most conserved regions of 25 sequences was performed. This analysis showed that *C. auris* Yps1 is related to *C. albicans* Sap9 and Sap10 (Figure 1A). CauYps1 showed a probable internal loop that could be removed similarly to ScYps1 by the cleavage of the monobasic or dibasic residues into its region (Figure 1B and Figure S2). On the other hand, *C. auris* Yps7 is associated with the clade of *C. glabrata* and *S. cerevisiae* Yps7. However, *C. glabrata* and *S. cerevisiae* did not show well-conserved aspartic and disulfide bridges (Figure 1D). Moreover, *C. auris* Yps3, Yps4, and Yps6 were less related to yapsin proteins, while *C. auris* Yps2 and Yps5 showed no relation to the yapsins included in the analysis (Figure 1A). The *CauYPS1–CauYPS7* genes are distributed across different chromosomes in the *C. auris* genome (Figure 1C). Although *CauYPS2* and *CauYPS5* are on the same chromosome, there is no phylogenetic relationship between them (Figure 1 and Figure S1).

### 3.3. The C. auris Proteins Yps1 and Yps7 Present a Secondary and Tertiary Structure of Aspartyl Proteases, Capable of Binding to the Specific Inhibitor Pepstatin A and Interacting with Other Proteins

The secondary and tertiary structures of mature CauYps1 and CauYps7 were predicted, as mentioned in Section 2. The most conserved regions of the yapsins showed a secondary structure similar to the templates *C. tropicalis* Sap and *C. albicans* Sap5 (Figure S3). In the case of the 3D structure, the templates selected were *C. tropicalis* Sap and *C. albicans* Sap1, which showed an identity and similarity of 33.5% and 52.7% with Yps1, and 18.2% and 33.1% with Yps7, respectively. The quality of the predicted tertiary structures of CauYps1 and CauYps7, suggested that they are suitable models. The percentage of residues in most favored regions in the Ramachandran plot, the overall quality factor (from ERRAT), and the percentage of residues showing an averaged 3D/1D score ≥ 0.1 (from VERIFY3D) correspond to 81.9%, 77.86%, and 90.07% for CauYps1 and 88.7%, 81.68%, and 50.85% for CauYps7.

**Figure 2:**
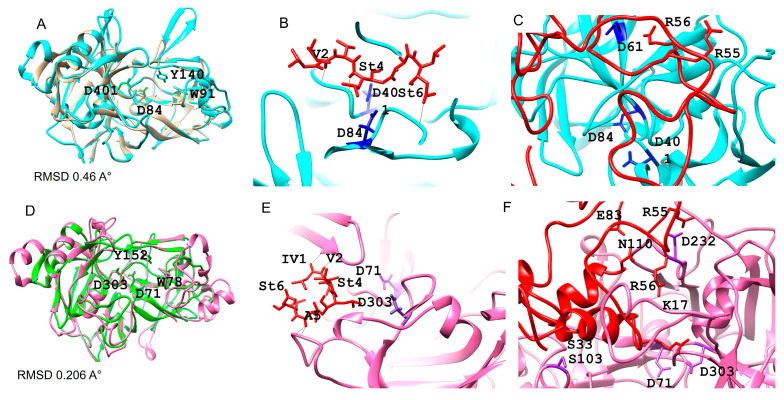
The predicted tertiary structure of the *C. auris* Yps1 and Yps7 and their interaction with the inhibitor pepstatin A and a synthetic substrate. (**A**,**D**) Overlap of the mature CauYps1 (cyan) and the *C. tropicalis* Sap (1J71) (brown), and CauYps7 (pink) and the *C. albicans* Sap1 (2QZW). Molecular docking of the CauYps1 (**B**) and CauYps7 (**E**) with pepstatin A (red), and (**C**,**F**) with a derived peptide of proinsulin (red), respectively. The catalytic residues Asp84 and Asp401 of CauYps1 interact with statines 4 and 6 of the inhibitor and Asp61 with Arg56 of the substrate. In contrast, the residue Asp232 of CauYps7 interacts with the statine 4 of the inhibitor and Arg55, Glu83, Gly84, and Asn110 of the substrate. Amino acid numbers are given based on the complete protein or peptide sequences.

Both protein structures of CauYps1 and CauYps7 exhibited a bilobed arrangement, forming a catalytic pocket. Each lobe contains one of the two catalytic aspartic residues, both of which are oriented toward the pocket. The molecular docking showed that pepstatin A (a hexapeptide inhibitor of aspartyl protease) fits into the pocket of the predicted structures. The binding energy of pepstatin A for Yps1 was −6.5 kJ/mol, while, for Yps7, it was −6.9 kJ/mol. Furthermore, the catalytic aspartic residues bind to the statine amino acid of pepstatin A and some amino acids of the proinsulin peptide (Figure 2).

In addition to proinsulin-derived peptide, sequences of homologous proteins to the targets of Yps1 of *S. cerevisiae* and Sap9 of *C. albicans* were identified in *C. auris*. The GPI-anchored cell wall putative proteins Utr2, Gas1, and Msb2, the first two involved in cell wall homeostasis, and the latter acts as a stress sensor, showed conserved monobasic (K or R) and dibasic (KR or RR) cleavage sites. Furthermore, the cell wall CauPir1 protein could be processed by Yps1 at the propeptide in a similar fashion to ScPir1; however, CauPir1 lacks the processing sites present in the internal repeats of ScPir1, whose function is related to cell wall stability through the linkage of the repeats to glucans. Additionally, the putative Matα precursor exhibited well-conserved KR sites, which were processed by Kex2 and, in its absence, by Yps1 (Figure S4).

### 3.4. Growth of C. auris CJ97 and 20-1498 in the Presence of Different Stressor Compounds

**Figure 3:**
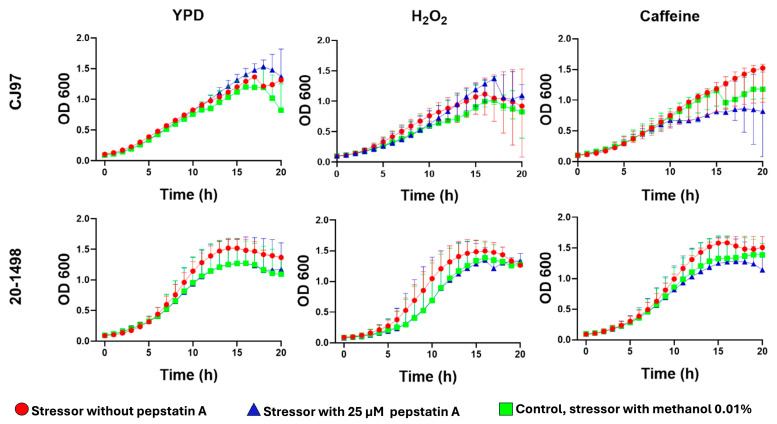
Impact of pepstatin A on the growth of *C. auris* strains CJ97 (clade III) and 20-1498 (clade IV). Yeast cultures were grown in YPD medium alone or supplemented with 10 mM H_2_O_2_ or 12 mM caffeine, either without pepstatin A or with 25 μM pepstatin A. Each stressor plus 0.01% methanol, the solvent used for pepstatin A, served as a control in addition to YPD. Experiments were conducted in triplicate, and error bars represent standard deviation.

*C. auris* yeast were cultured for 20 h at 37 °C in the presence of H_2_O_2_, caffeine, NaCl, and SDS without or with pepstatin A to determine whether aspartyl protease inhibition affected yeast growth. The results showed that H_2_O_2_, NaCl, and SDS inhibit the growth of *C. auris*. When pepstatin A was also present in media containing H_2_O_2_ and caffeine, it exhibited a subtle but sustained effect on the growth of the yeasts compared to the controls (without pepstatin A) (Figure 3). In contrast, *C. auris* strains grown in media with pepstatin plus SDS and NaCl did not exhibit changes in growth compared to the controls (Figure S3).

### 3.5. Differential Expression of YPS1 and YPS7 Genes Under Different Stress Conditions

**Figure 4:**
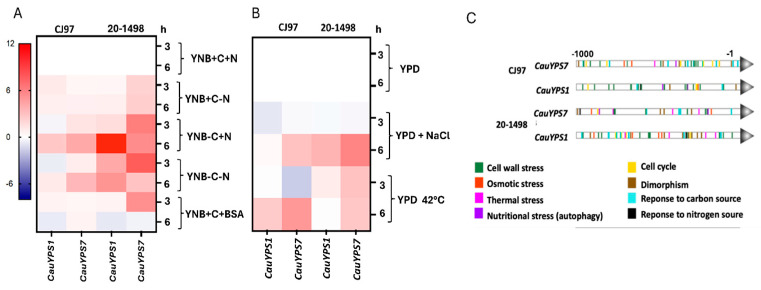
Differential expression of *CauYPS1* and *CauYPS7* genes of the strains CJ97 (clade III) and 20-1498 (clade IV). Heatmap representing the log2 of the gene expression change determined by RT-qPCR. (**A**) RNA extracted from yeast after 3 and 6 h of treatment in YNB medium supplemented with +C = 2% dextrose (as carbon source), +N = 0.5% ammonium sulfate, or +BSA = 0.5% BSA (as nitrogen source). Negative signal indicates the absence of the corresponding stimulus. (**B**) RNA extracted from yeast after 3 and 6 h of treatment at 37 °C in YPD medium without or with 1.5 M NaCl (-NaCl and +NaCl, respectively) or YPD at 42 °C. Two independent experiments were carried out in triplicate. (**C**) Predicted TFBSs into the 1000 base pairs upstream of the *CauYPS1* and *CauYPS7* genes using the YEASTRACT database.

Yeasts of *C. auris* from clades III and IV were grown in YPD to the early stationary phase and then subjected to different nutritional conditions, 1.5 mM NaCl, and at 42 °C. Gene expression was conducted 3 and 6 h after each stimulus. The results were grouped in a heatmap according to the expression levels of genes (Figure 4). It was observed that the two genes of the CJ97 and 20-1498 strains were differentially expressed. *CauYPS1* of both strains was overexpressed mainly after 6 h without a carbon source compared to the complete medium. The *CauYPS7* of 20-1498 (clade IV) showed a higher expression than the *CauYPS7* of the CJ97 strain (clade III) after 3 and 6 h of carbon and/or nitrogen starvation. Instead of ammonium sulfate as a nitrogen source, BSA induced the *CauYPS7* of 20-1498 after 3 h, while, after 6 h, the *CauYPS1* and *CauYPS7* of both strains were repressed.

In general, the *CauYPS1* and *YPS7* genes of both strains were overexpressed after 6 h at 42 °C and with NaCl compared to YPD at 37 °C, and the expression of *CauYPS7* of the 20-1498 strain was usually higher than that of CJ97. On the other hand, *CauYPS1* and *YPS7* showed repression after treating yeasts of the CJ97 and 20-1498 with NaCl for 3 h (Figure 4A,B).

The expression levels of the *C. auris YPS1* and *YPS7* genes agreed with their theoretical TFBSs found 1000 bp upstream of the encoding regions. These sites could bind transcription factors linked to, for example, the response to the cell wall, osmotic and thermal stress, and nutritional stress. Moreover, *CauYPS1* and *CauYPS7* showed differences in the disposition of the TFBSs along the analyzed upstream regions between the strains (Figure 4C).

### 3.6. Transcriptional Response of C. auris Strain 20-1498 Under Heat Stress

Given the overexpression of *CauYPS1* and *CauYPS7* detected by RT-qPCR under heat stress conditions, we performed a transcriptomic analysis of the first Mexican isolate of *C. auris* (strain 20-1498) to identify genes involved in the heat stress response. The total RNA was extracted from stationary-phase cultures grown in nutrient-rich medium (YNB supplemented with carbon and nitrogen sources) and incubated for six hours at either 37 °C (control) or 42 °C, using three biological replicates per condition.

A sample distance matrix and three-dimensional principal component analysis (PCA 3D) revealed a clear separation of samples according to incubation temperature, indicating a high reproducibility among replicates and distinct transcriptional profiles between conditions (Figure S5).

**Figure 5:**
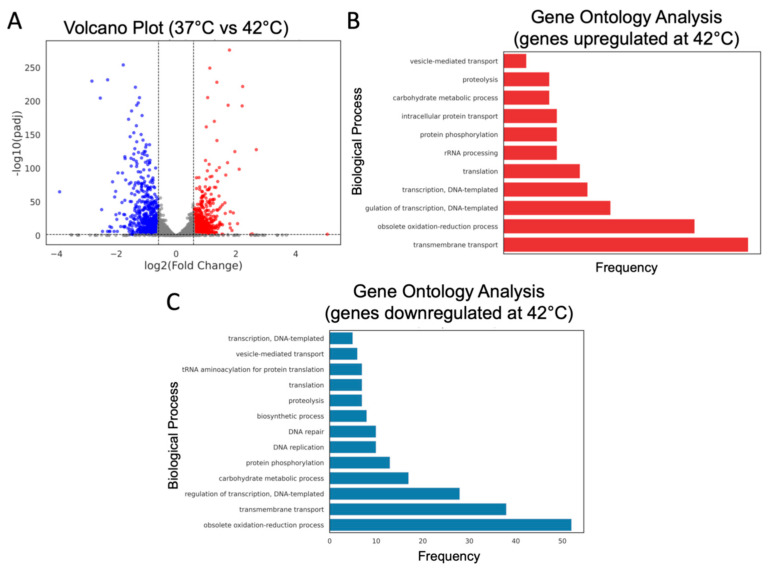
Differentially expressed genes and biological process ontology in *C. auris* under heat stress. (**A**) Volcano plot of differentially expressed genes. A threshold of log2FoldChange ≥ 0.585 or ≤−0.585 along with an adjusted *p*-value (*padj*) < 0.05 was applied. Downregulated genes are shown in blue, upregulated genes in red, and genes with no significant change in gray. (**B**,**C**) Functional enrichment analysis of GO terms for upregulated (**B**) and downregulated (**C**) genes at 42 °C.

Applying cutoffs of log2FoldChange ≥ 0.585 or ≤−0.585 and an adjusted *p*-value (*padj*) < 0.05, we identified 1341 differentially expressed genes (DEGs) out of 5538 mapped genes, with 630 upregulated and 711 downregulated under heat stress (Figure 5A). A functional enrichment analysis of these DEGs indicated the activation of pathways related to transmembrane transport, redox processes, proteolysis, and phosphorylation, whereas genes associated with cellular component synthesis, DNA repair and replication, and vesicular transport were downregulated under these conditions (Figure 5B,C).

**Figure 6:**
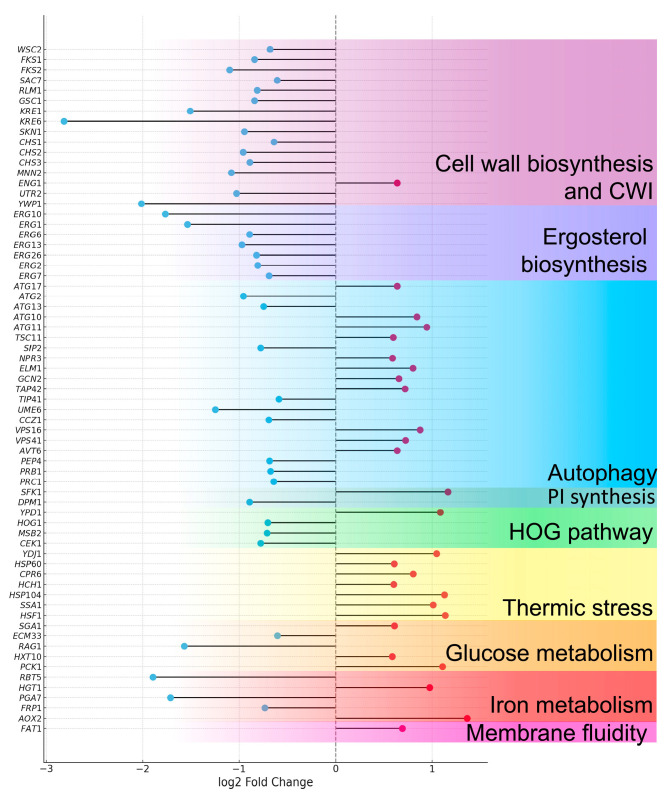
Lollipop plot of differentially expressed genes in *C. auris* under heat stress conditions. Strain 20-1498 (clade IV) was cultured in YNB+C+N medium at 37 °C and 42 °C. A threshold of log2FoldChange ≥ 0.585 or ≤−0.585 with an adjusted *p*-value (*padj*) < 0.05 was applied. Genes are arranged in order of their functional relevance. Red circles indicate upregulated genes, while blue circles correspond to downregulated genes.

Additionally, orthologous genes from *C. albicans* were identified within the set of differentially expressed genes in *C. auris* through a pBLAST analysis focused on specific biological pathways of interest. These included sterol, phosphatidylinositol, and cell wall biosynthesis, as well as stress response genes associated with the cell wall integrity pathway, heat-stress-induced autophagy, the HOG (High Osmolarity Glycerol) pathway, the calcineurin pathway, and other genes linked to thermal stress (Figure 6, Table S1).

### 3.7. Effect of Pepstatin A on the Microscopic Morphology of C. auris 20-1498

*C. auris* cells were cultured in YPD medium with 12.5, 25, and 50 μM of pepstatin A, and microscopic morphology was observed using scanning electron microscopy (SEM). Cells without the inhibitor plus 0.01% methanol or not were used as a control. In the control conditions, oval-shaped yeasts of approximately 2–2.5 × 3–4 μm were observed with some budding and smooth surfaces. At 12.5 μM, yeasts with a rough appearance were observed at a percentage of 3.5%. This effect was dose-dependent, as yeasts with this morphology increased to 4.5% at 25 μM and 6.5% at 50 μM of pepstatin A. At the highest concentration, concave-looking cells were also observed in 60%.

**Figure 7:**
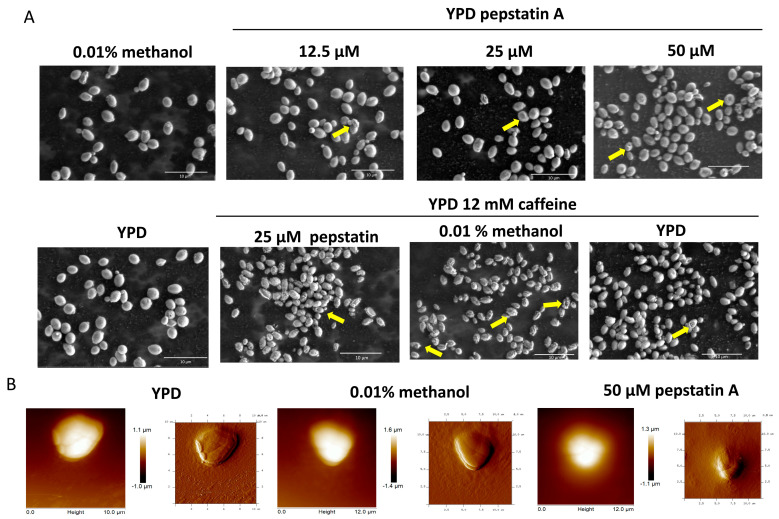
Effect of pepstatin A on the microscopic morphology of *C. auris* 20-1498. (**A**) SEM micrographs showing cells with rough and concave surfaces (arrows) after being grown for 22 h in YPD supplemented with pepstatin A and/or caffeine at the indicated concentration. YPD and YPD with methanol are the control conditions. Images of the fixed cells were acquired using an SEM microscope Quanta FEG 250 (FEI Company, Eindhoven, The Netherlands). (**B**) Height (left) and amplitude (right) images showing the yeast surface of cells grown as mentioned before and treated for AFM observations as indicated in Section 2. Images were acquired with an atomic force microscope.

A synergistic effect was observed when cells were grown in YPD with 25 μM pepstatin A and 12 mM caffeine. Nearly 90% of the cells showed evident damage to the cell wall, with a wrinkled appearance and presumptive holes, in contrast to the yeasts grown in the medium with only caffeine or pepstatin (Figure 7A).

The effect of pepstatin A on the cell surface topography of *C. auris* was also evaluated by atomic force microscopy (AFM). The roughness of cells with pepstatin A 50 μM increased in contrast to cell growth in control conditions (Figure 7B). The AFM analysis suggests a possible decrease in the size of *C. auris* yeasts when treated with pepstatin A. The length of the yeast would be affected, but not the height. The length varies from 4.2–4.7 μm of yeast in the control condition to 3.7–3.9 μm of yeast treated with pepstatin (Figure 7B).

## 4. Discussion

*C. auris* has emerged as a multidrug-resistant pathogen associated with intra-hospital outbreaks that cause systemic infections with high mortality, becoming a serious public health problem worldwide. This yeast is distinguished from other human pathogenic species by its thermo- and osmotolerance, characteristics linked to its ability to survive and persist in various harsh natural and healthcare facility environments, as well as on the human skin. Combined with the effects of climate change and possibly the excessive use of antifungals, these traits could have contributed to the recent and simultaneous emergence of this yeast as a pathogen in several regions of the world [15,16,17].

Among other characteristics that distinguish *C. auris* from other pathogenic yeasts are its capacity to form a multilayered biofilm, to cause invasive infections without forming mycelium, and immune evasion, features contributing to virulence and attributed in part to the yeast cell wall [19]. The use of this structure as a helpful target to control fungal infections has been widely discussed, and, because of their contribution to cell wall integrity, GPI-anchored cell wall proteins have been studied for this purpose. The APX001A is a novel agent that inhibits fungal Gwt1 protein, an enzyme involved in the synthesis of the glycosylphosphatidylinositol moiety of GPI-anchored proteins, and the treatment of *C. auris* with this compound impairs the maturation and localization of GPI proteins, thereby compromising cell viability [19,44].

Fungal yapsins are extracellular aspartyl proteases GPI-anchored to the yeast cell wall or membrane. These enzymes play a role in nutrient acquisition, dimorphism, and cell wall integrity, accounting for the virulence in pathogenic fungi [23,45]. Here, we identified seven genes encoding putative yapsins in the genome of *C. auris* B11220 (clade II), which were distributed in four chromosomes. In contrast to the multigene family of *C. glabrata* yapsin, the genes of *C. auris* did not exhibit a tandem arrangement, suggesting recent duplication [3]. The putative yapsins of *C. auris* range in size from 372 to 696 amino acids, suggesting potential functional variability among them, typical of this multigene family [3,23]. The seven yapsin-encoding sequences were also identified in the genomes of the CJ97 (clade III) and 20-1498 (clade IV) *C. auris* strains, which were practically identical between strains, except for putative CauYps5, which is shorter in B11220 than in CJ97 and 20-1498 (from 697 in the aforementioned strain to 703 amino acids in the two latter ones). Most of these amino acid sequences exhibited typical characteristics of yapsin-type aspartyl proteases [9,46]: the two conserved aspartic residues within the catalytic domain, except for Yps2 and Yps5, the cysteines responsible for disulfide bridge formation, the signal peptide, propeptides with dibasic or basic sites processed by Kex2 or autoprocessed, and the omega site characteristic of GPI-anchored proteins [2,5,46,47]. The predicted CauYps2 and CauYps5 do not present the aspartic catalytic residues in the consensus sequence, in contrast to yapsins of *S. cerevisiae* and *C. glabrata*; the exception is the CgYps7 [3,48]. In addition, the phylogenetic analysis showed that CauYps2 and CauYps5 are not related to the canonical yapsins of *S. cerevisiae*, *C. glabrata,* and *C. albicans*. Particularly, putative CauYps1 clustered with the Sap9 and Sap10 of *C. albicans* and the CauYps7 to *S. cerevisiae* and *C. glabrata* Yps7 proteins. It suggests that *YPS1* and *YPS7* of *C. auris* are homologous to the *YPS1*, *SAP9*, and *YPS7* of *S. cerevisiae* and *C. albicans*, in agreement with the situation previously described by Alvarado et al. [20].

In addition, CauYps1 and CauYps7 also showed similar domains to those described for the Yps1, Sap9, and Yps7 of other yeasts. Notably, *C. auris* Yps1 showed an internal loop like that of the Sap9 and Yps1 of *C. albicans* and *S. cerevisiae*, proteins where this region undergoes autoprocessing, generating two subunits linked through a disulfide bridge [2]. Dibasic sites upstream of the ω site seem to contribute to the shedding activity of the Yps1 of *S. cerevisiae*, acting on itself and under other GPI-anchored proteins such as Gas1, Utr2, and Msb2 [2,49]. Moreover, the conserved regions of Yps1 and Yps7 with SAPs of *C. albicans* showed moderate identity percentages. Nevertheless, they are considered adequate for generating predictive models in structural biology, especially for medically relevant proteins such as aspartic proteases [50]. The secondary and tertiary structures like the A1 family of aspartyl protease showed two non-identical lobes, each one containing one of the two catalytic aspartic residues, and the flap, a loop part of the S1 subsite [10], and their catalytic pockets interact through their aspartic catalytic residues with pepstatin A and other residues with proinsulin-delivered peptide containing mono or dibasic sites. The above observations suggest that *C. auris* yapsins are potential targets of this inhibitor peptide, such as the yapsin of *S. cerevisiae*, *Aspergillus oryzae*, and *C. albicans*, besides the affinity of these yapsins for substrates rich in basic residues [4,48,51].

Since the theoretical analysis points to the probable inhibition of at least CauYps1 and CauYps7 by pepstatin A, the effect of this inhibitor on the growth and the superficial structures of *C. auris* of the clades III and IV was evaluated to provide insights into the role of yapsins. Pepstatin A is a known inhibitor of aspartic proteases and has helped investigate the fungal protease function during morphogenesis, and growth in stress conditions, and in superficial structure morphology [52,53]. In the study conditions, the growth of *C. auris* in media containing pepstatin A was not affected, despite other aspartic proteases, such as the previously described SAPs or the vacuolar PrA, being inhibited by pepstatin [22,53]. Furthermore, pepstatin was tested in concert with H_2_O_2_, caffeine, NaCl, or SDS, stress conditions, where yapsins of other yeasts seem to be involved in maintaining the cell wall homeostasis [3,23]. Although all the compounds inhibited the growth of both the clade III and IV *C. auris* strains, in a similar behavior as observed before [54], together with pepstatin, only peroxide and caffeine influenced the growth of the yeast, suggesting that aspartic proteases may be involved in *C. auris*’s handling of stress associated with the cell wall. Thus, *C. auris* 20-1498 of the clade IV was subjected to SEM and AFM; while the former has been widely used to observe the superficial structures of yeast, the latter has been used more recently for this purpose with other yeasts, showing its utility [55]. Under control conditions, yeast cells exhibited a typical oval morphology with budding evidence. However, as the concentration of the inhibitor increased, progressive changes in cell morphology were observed, from a rough appearance to cells with a concave shape at higher concentrations, which increased further when caffeine was added. All of this supports the idea that cell wall integrity depends partially on the activity of aspartic proteases, as has been shown in *P. brasiliensis* and *C. glabrata*. In the latter, using a strain carrying an inactive Yps1^D91A^, it was evidenced that active yapsins are required for cell wall homeostasis [47,52]. Although, now, it is not possible to assign this result to a specific aspartyl protease, similar behavior on the cell wall was observed in *C. glabrata* by SEM, as a consequence of the deletion of their 11 yapsins [56], and at least the Sap9 of *C. auris*, here called CauYps1, seem to be involved in the biofilm formation through its regulation by the HOG pathway [57].

To relate the function of *C. auris* yapsins to the stress response of the yeast, the expression of the genes *YPS1* and *YPS7* was evaluated during nutrient scarcity, high temperature, and the presence of NaCl, which also activate the response of the cell through the cell wall integrity response [58]. The analysis showed that, while the lack of carbon sources induces the expression of *YPS1* of both strains CJ97 (clade III) and 20-1498 (Clade IV), the lack of nitrogen source or the presence of BSA induced mainly that of the *YPS7* gene of the 20-1498 strain. These results agree with previous reports on the regulation of gene expression encoding yapsins 1 and 7 of *C. glabrata* in response to dextrose and nitrogen depletion [24,59]. Moreover, *S. cerevisiae YPS7* is induced by alternate carbon sources such as mucin, a polysaccharide encountered in the host cell [60]. Osmotic stress conditions (NaCl) and thermal stress (42 °C) also regulate the expression of *CauYPS1* and *CauYPS7*, as observed before in *C. glabrata*. In the late case, authors suggested that different clinical strains showed a differential expression of *CgYPS1* and *CgYPS7,* which may be a consequence of the differences in promoter gene sequences [59]. The prediction of TFBSs 1000 bp upstream of the coding sequences of the *YPS1* and *YPS7* genes of both strains displayed dissimilar arrangements. However, these promoters may be regulated by the CWI, HOG, and calcineurin pathways implicated in membrane and cell wall stability [61]. The relatively mild change in expression, mainly of *CauYPS1* under these conditions, could be a result of the suggested efficient homeostatic mechanisms that allow *C. auris* to maintain its physiology without the need for a massive transcriptional response, which may be related to post-translational regulation or epigenetic mechanisms [61].

Given the predominantly *CauYPS1* and *CauYPS7* behavior, and considering the thermotolerance of *C. auris*, we assessed the transcriptomic profile of *C. auris* 20-1498 after 6 h at 42 °C compared to 37 °C. This analysis showed that genes such as *HSF1* and *HSP90,* as well as others that influence the response to heat stress, are overexpressed, as previously described in other strains of *C. auris* and other pathogenic yeasts [62]. Along with heat shock proteins, autophagy helps cells to maintain protein homeostasis after stress stimuli [63]. In this case, some autophagy genes are overexpressed, which could suggest a role in the degradation of misfolded proteins. Moreover, genes involved in iron homeostasis, and glucose and pyruvate metabolism are upregulated, as has been reported for this yeast [62]. Moreover, genes encoding proteins that regulate membrane fluidity showed an increased expression, as observed in other fungi [64]. On the other hand, the *HOG1* gene was downregulated at 42 °C in contrast to 37 °C, in agreement with the results recently reported by Xiao et al. [62]. Furthermore, genes involved in the synthesis of chitin and β-glucans are downregulated, as well as cell surface proteins that are involved in the remodeling of the *C. albicans* cell wall at 42 °C [65]. It is worth noting that, in contrast to *C. albicans*, *C. auris* exhibits a lower chitin content, which is likely related to its cell wall flexibility, allowing the yeast to adapt to osmotic stress [21].

**Figure 8:**
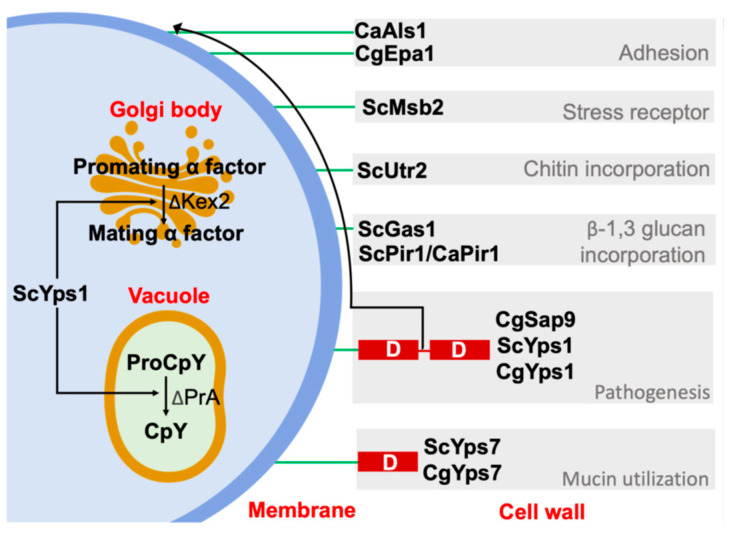
Functions of Yps1 and Yps7 of yeast *S. cerevisiae* (Sc), *C. albicans* (Ca), and *C. glabrata* (Cg). The yapsins appear to be redundant to carboxypeptidase Kex2; both cleave monobasic or dibasic residues. Furthermore, a sheddase activity of yapsins on cell wall proteins and itself could be related to cell wall homeostasis, and other functions shown in the figure. GPI-anchor: green line. [3,4,5,43,56,66,67,68]. Figure generated by the authors.

The role of yapsins in cell wall homeostasis could be due to their ability to release cell wall proteins, since these proteins contain similar dibasic or monobasic residues recognized by this protease and by Kex2, as previously suggested [48]. For example, GPI-anchored the proteins Gas1 (Phr family), Utr2 (Chr family), and Msb2, which are a β-1,3-glucan transferase and chitin transferase, respectively, both of which are involved in conveying these moieties to the growing β-1,3-glucans, allowing the cross-linking of the cell wall. On the other hand, Msb2, a transmembrane protein that regulates the MAPK pathway in response mainly to nutrient scarcity (glucose) and osmotic stress, is activated after the removal of the extracellular HMD domain by Yps1 action on the cleavage domain (CD) [49,66,67,68]. Furthermore, proteins as the soluble cell wall protein Pir1 of *Saccharomyces* and *C. albicans* are cleaved by Yps1 and Sap9 and Sap10, respectively, between binding repeats, probably altering their binding toβ-1,3-glucans [4,49]. On the other hand, the α-mating factor (Matα), a natural target of Kex2, is processed by *S. cerevisiae* Yps1 in Δ*kex2* strains [5,46,47,68]. In contrast, in the double mutant Δ*erg6*Δ*pep4*, YPs1 processes the carboxypeptidase Y (CpY) to the mature form CPY, suggesting a role in vacuole homeostasis, similar to the yapsins of *C. glabrata* [56,68,69]. Other proteins released by yapsins are the adhesins Epa1 and Als1 of *C. glabrata* and *C. albicans*, respectively. All of this, together with the suggested capacity of yapsins to degrade host proteins, could be relevant in their role as virulence factors [3,4] (Figure 8).

Thus, the *C. auris* genome holds five genes encoding well-conserved putative extracellular yapsins that may be anchored to the membrane or cell wall. Two are less related to aspartyl proteases. Nevertheless, some aspartic proteases with an alanine residue instead of the catalytic aspartic acid have been described [10]. Using pepstatin A, an inhibitor of aspartyl proteases, the growth of both strains of *C. auris* was slightly and differentially affected, mainly in the presence of compounds that induce the cell wall integrity pathway, such as H_2_O_2_ and caffeine, the latter through the TOR pathway [70,71]. Moreover, after caffeine with pepstatin A, the cell wall became rough with depressions. These results suggest a role of aspartic proteases in maintaining cell wall integrity during stress conditions. Other well-known conditions that induce the reorganization of the cell wall of *Candida* spp. included nutrient, temperature, and osmotic stress [58], conditions that differentially regulate the *CauYPS1* and *CauYPS7* expression between the tested strains, showing a specific clade transcriptome response. Jenull et al. [61] suggest that the transcriptional profile of *C. auris* associates with phenotypic traits of the strains of each clade. Moreover, the transcriptomic profile of the strain 20-1498 (clade IV) showed an increased expression of genes related to chaperone function, glucose and iron homeostasis, and membrane fluidity, as previously shown [62]. In addition, autophagy genes (*ATGs)* are also overexpressed, suggesting a role in the removal of damaged proteins. Thus, as in other fungi, the TOR pathway could be a crosstalk signaling of stress conditions to regulate the cell wall remodeling and the vacuole function [70], where yapsin Yps1 and CauYps7 might play a role, through the processing of proteins to mature or release them from the cell wall or membrane.

In conclusion, the results of pepstatin A inhibition on cell wall morphology, growth, and *YPS1* and *YPS7* gene expression suggest that the yapsins CauYps1 and CauYps7 may play a role in *C. auris* cell wall integrity under stress conditions. However, further efforts are needed in order to obtain corresponding mutants lacking the corresponding enzymes and to evaluate the role of the other yapsins. *C. auris* yapsins could be a therapeutic target for the design of new antifungal or antivirulence agents.

## Data Availability

The original contributions presented in this study are included in the article/Supplementary Materials. Further inquiries can be directed to the corresponding authors.

## Supplementary Materials

The following supporting information can be downloaded at: https://www.mdpi.com/article/10.3390/jof11080573/s1, Figure S1: Characteristics of predicted yapsins of three *C. auris* strains of clades II, III, and IV; Figure S2: Predicted secondary structure and motifs of yeast yapsins; Figure S3: Effect of pepstatin A on the growth of *C. auris* in the presence of different compounds; Figure S4: Predicted protein targets of Yps1 of *C. auris*; Figure S5: Global transcriptomic analysis of *C. auris*. Strain 20-1498, belonging to clade IV, was cultured in YNB+C+N medium at 37 °C and 42 °C for 6 h [68,70]; Table S1: Accession numbers and data generated from the transcriptomic analysis of *C. auris* at 42 °C.
