# Supplementary material for: The GPI-Anchored Aspartyl Proteases Encoded by the YPS1 and YPS7 Genes of Candidozyma auris and Their Role Under Stress Conditions
Source: J Fungi (Basel). 2025 Aug 1;11(8):573. doi: 10.3390/jof11080573 (PMC12387857; doi:10.3390/jof11080573)
Supplement: Supplementary file 1 [file jof-11-00573-s001.zip › jof-3740372-supplementary.pdf]

Supplementary material

A

	1	2	3	4	5	6	7	8	9	10	11	12	13	14	15	16	17	18	19	20	21
1. CauYps1 III		98.8	98.5	28.9	28.8	28.9	31.8	32.2	32.7	29.9	30.2	29.9	32.6	23.9	21.5	21.1	21.1	20.4	19.3	19.3	20.1
2. CauYps1 II	99.3		98.0	29.1	29.1	28.8	32.1	32.2	33.0	30.6	30.9	30.6	32.8	24.1	20.9	20.9	20.9	20.2	19.5	19.5	19.8
3. CauYps1 IV	99.2	98.8		29.1	28.9	29.1	31.7	32.4	32.6	30.1	30.4	30.1	32.6	23.4	21.6	20.9	20.9	20.1	19.8	19.8	20.3
4. CauYps2 III	49.6	50.0	49.7		96.4	98.7	30.6	29.9	31.1	31.0	31.4	32.2	39.0	31.8	27.4	20.3	20.3	20.4	20.4	20.4	19.5
5. CauYps2 IV	48.4	48.1	48.5	97.2		97.6	30.1	29.8	30.3	31.0	31.4	32.2	38.6	31.4	27.2	20.1	20.1	20.9	19.9	19.9	20.6
6. CauYps2 II	49.4	48.6	49.5	99.1	98.1		30.5	30.1	30.9	31.0	31.4	32.2	38.6	31.4	26.7	20.3	20.3	20.4	20.1	20.1	19.6
7. CauYps3 III	49.6	49.2	49.4	45.9	45.1	45.5		95.6	96.3	32.7	32.7	32.5	35.9	26.2	25.0	17.6	17.6	18.3	20.0	20.0	20.3
8. CauYps3 IV	48.1	47.7	48.1	46.2	45.9	46.1	97.0		96.4	32.0	32.0	32.0	35.6	25.7	24.8	17.3	17.3	17.7	19.9	19.9	20.4
9. CauYps3 II	49.7	49.3	49.6	47.3	46.1	46.8	97.9	96.9		32.1	32.1	32.0	36.4	26.7	24.9	18.1	18.1	18.3	21.1	21.1	21.2
10. CauYps4 III	50.6	50.7	50.2	51.6	50.5	50.7	48.6	46.8	47.4		99.8	98.6	33.1	25.0	23.3	21.2	21.2	20.3	22.2	22.2	22.7
11. CauYps4 II	50.9	51.0	50.5	51.9	50.8	51.0	48.8	47.4	47.6	99.8		98.7	32.9	25.0	23.3	21.4	21.4	20.8	22.0	22.0	22.6
12. CauYps4 IV	50.4	50.3	50.0	51.7	50.4	50.8	49.1	47.7	47.8	99.5	99.6		33.4	25.4	23.7	21.9	21.9	21.1	22.1	22.1	22.8
13. CauYps5 III	52.1	52.0	51.9	59.1	59.3	59.3	49.9	50.2	50.3	53.2	53.0	53.1		68.8	51.4	20.0	20.0	21.0	24.1	24.1	23.3
14. CauYps5 II	37.9	37.8	37.5	46.0	45.6	45.8	35.8	36.0	36.2	38.5	38.6	38.6	69.9		51.6	19.1	19.1	19.2	20.4	20.4	19.7
15. CauYps5 IV	41.6	40.8	41.2	45.6	47.1	45.4	39.7	39.5	40.1	39.6	39.7	39.9	64.8	58.3		18.1	18.1	17.9	19.1	19.1	18.8
16. CauYps6 III	39.4	39.7	39.3	37.5	36.2	37.3	30.4	30.4	31.0	36.4	37.0	38.6	36.2	37.2	34.8		100.0	96.6	20.2	20.2	17.2
17. CauYps6 II	39.4	39.7	39.3	37.5	36.2	37.3	30.4	30.4	31.0	36.4	37.0	38.6	36.2	37.2	34.8	100.0		96.6	20.2	20.2	17.2
18. CauYps6 IV	39.2	39.8	38.6	37.7	38.5	37.5	31.2	29.3	30.9	36.7	37.7	37.7	37.7	37.7	33.7	97.3	97.3		19.4	19.4	18.7
19. CauYps7 II	41.4	41.4	41.9	38.8	39.9	38.8	36.8	36.4	37.9	42.1	41.9	44.2	41.7	33.5	36.0	35.8	35.8	37.1		100.0	96.9
20. CauYps7 III	41.4	41.4	41.9	38.8	39.9	38.8	36.8	36.4	37.9	42.1	41.9	44.2	41.7	33.5	36.0	35.8	35.8	37.1	100.0		96.9
21. CauYps7 IV	40.2	40.0	40.2	39.6	40.3	39.4	37.0	37.4	38.1	41.2	41.1	44.2	41.2	32.6	36.0	36.9	36.9	36.5	97.1	97.1	

B

Protein	Strain	Protein length (aa)	SP	D1	D2	N-gly	ω	Mw (kDa)	*pI*
Yps1	B11220	590	1-21	84	402	7	566	62.94	4.84
	CJ97	589	1-21	84	401	7	565	62.94	4.81
	20-1498	590	1-21	84	402	7	566	63.14	4.89
Yps2	B11220	533	1-15*	-	322*	6	504	58.45	4.95
	CJ97	533	1-15*	-	322*	6	504	58.58	4.88
	20-1498	533	1-23*	-	322*	6	504	58.74	5.09
Yps3	B11220	696	1-22	71	428	5	688	77.21	4.82
	CJ97	703	1-22	71	435	5	675	77.92	4.99
	20-1498	703	1-22	71	435	5	675	78.44	4.84
Yps4	B11220	559	1-16	80	344	9	536	59.85	4.68
	CJ97	558	1-16	80	344	9	536	59.64	4.68
	20-1498	559	1-16	80	344	9	536	59.88	4.65
Yps5	B11220	372	-	-	182*	4	346	40.25	4.88
	CJ97	528	1-15	73	338*	5	502	57.33	4.85
	20-1498	508	1-17	-	338*	5	482	57.92	9.35
Yps6	B11220	443	-	73	264	12	422	46.84	5.46
	CJ97	443	-	73	264	12	422	46.84	5.46
	20-1498	443	-	64	264	12	422	46.99	5.46
Yps7	B11220	556	1-18	71*	303	8	526	59.25	4.56
	CJ97	556	1-18	71*	303	8	526	59.25	4.56
	20-1498	556	1-18	71*	303	8	526	59.47	4.58

- The motive is not present

* The motive DT(S)G is not conserved

Figure S1. Characteristics of predicted yapsins of three *C. auris* strains of clades II, III and IV. **A)** Identity (blue) and similarity (green) (%) of sequences of the putative yapsins of *C. auris* B11220, CJ97 and 20-1498. Complete protein sequences (preproenzymes) were compared. **B)** Motifs found in the predicted yapsins of *C. auris*. SP: signal peptide, D1 and D2: catalytic aspartic residues, N-gly: number of glycosylation sites, ω: omega site of GPI anchorage; *pI*: isolectric point.

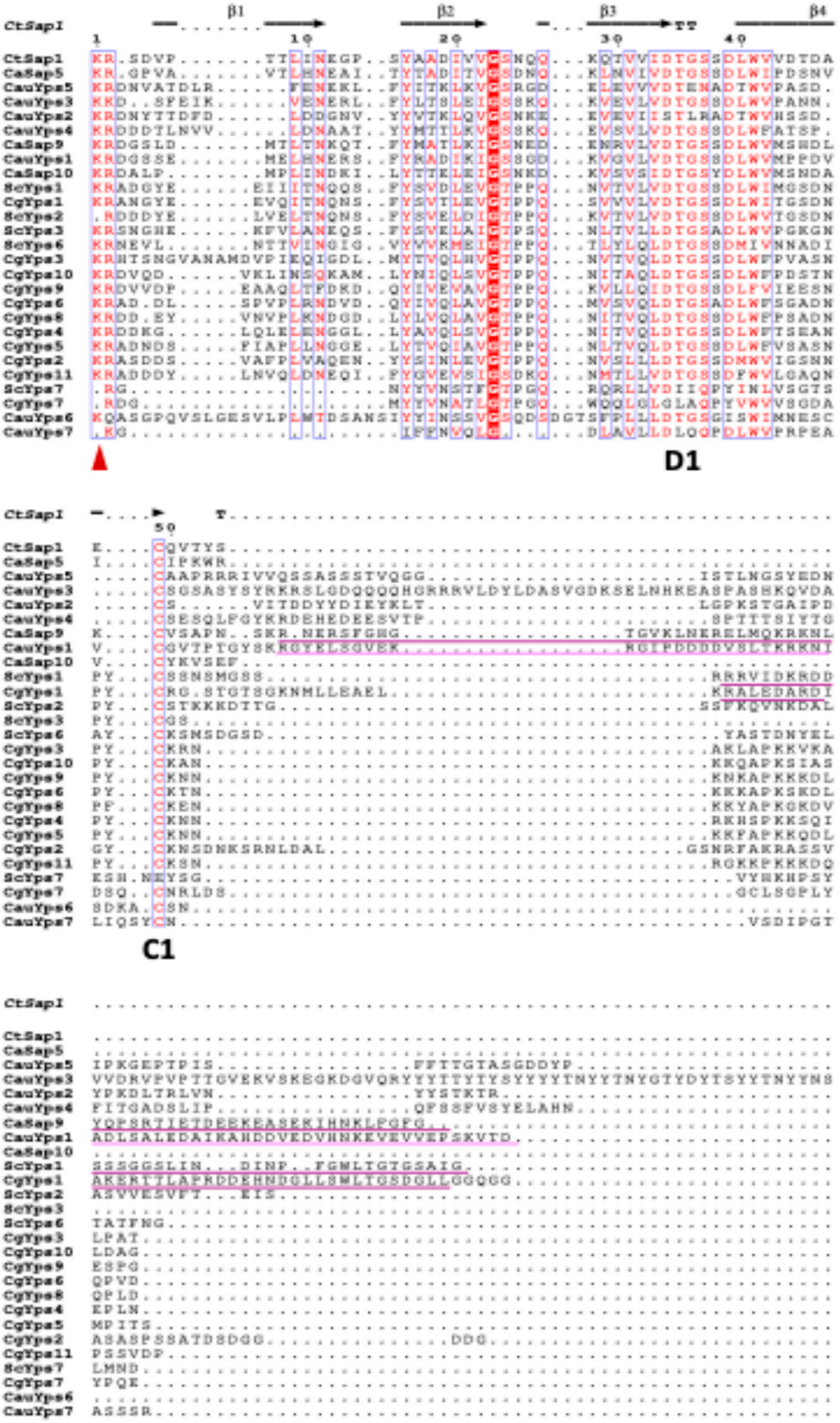
D1
C1

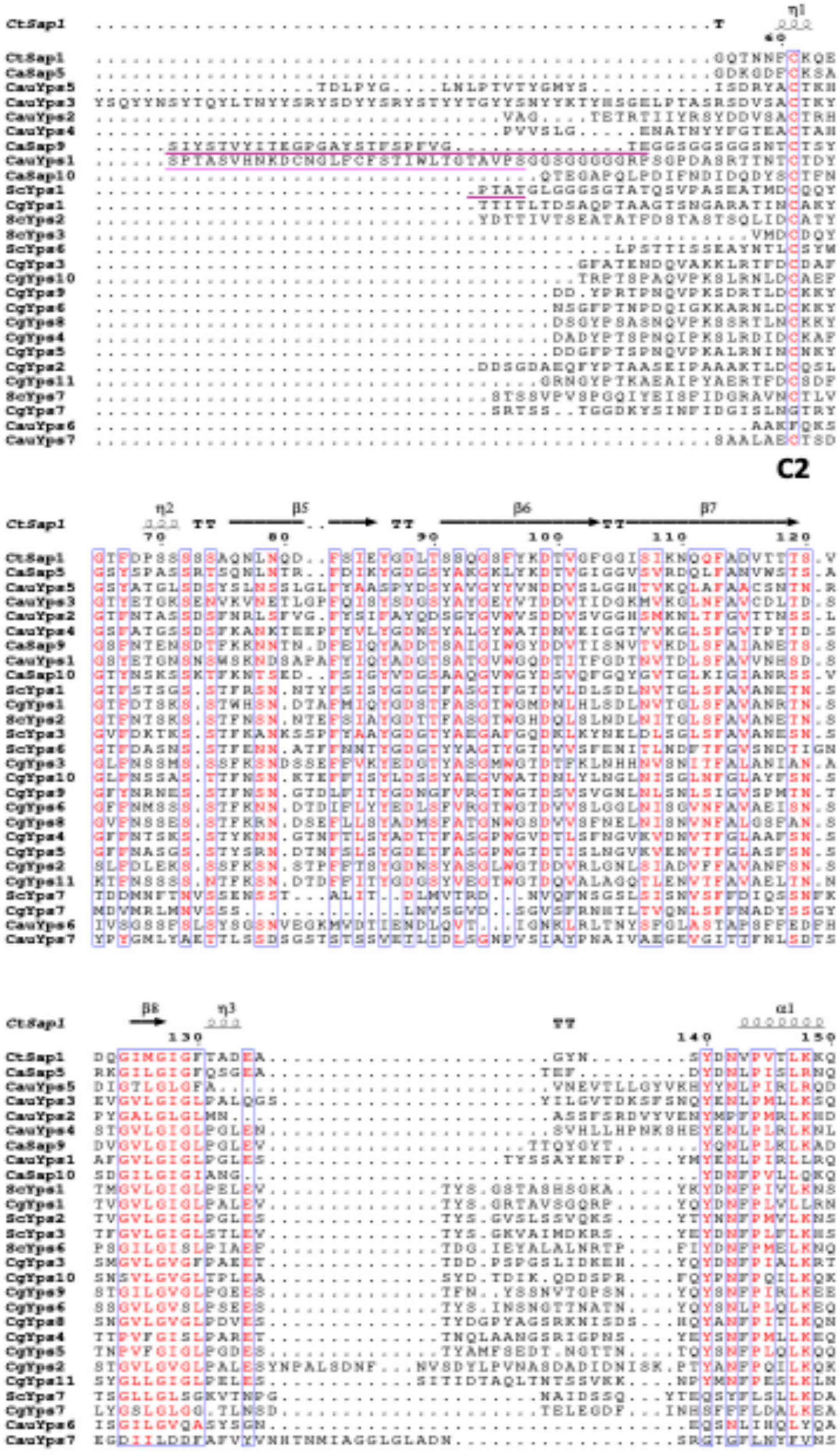
C2

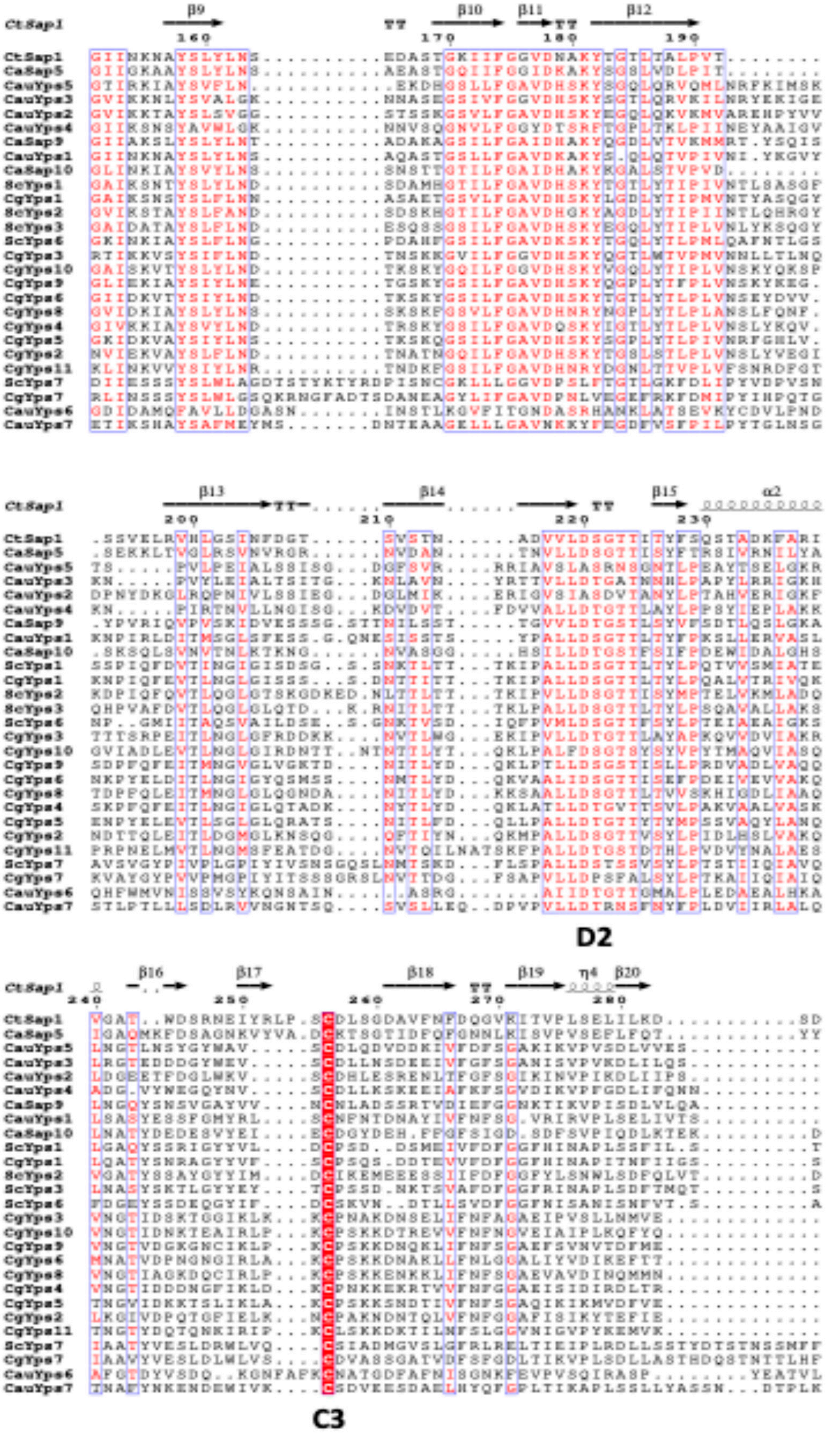

CtSap1
β9
β10
β11
β12
β13
β14
β15
α2
β16
β17
β18
β19
η4
β20
D2
C3

```
                 β21  β22          TT β23 α3    β24        β25
CtSap1           ---> --.>            --> oooo  ------->   -------->
                 290.          300.      310.      320.         330.

CtSap1     SS.....ICYFGIS.R......NDANILGDNFLRRAYIVYDLDDKTISLAQVKYT..SSS
CaSap5     TSGKPFPKCEVRIRE.......SEDNILGDNFLRSAYVVYNLDDKKISMAPVKYT..SES
CauYps5    ...ESGSSCFLNIRTD......EMFPSLGDSFLRNAYVVYDMEDYEVALAQAADGSSE..
CauYps3    ...VRGSYCYLGISED......NKTPSLGDSFLRNAYVVYDLENYEIALAQASDGSGE..
CauYps2    ...TSQKDCFLHMYPQ......EGQVYLGDSFLRNAYAVFDLENEEIALAQAAHGVRE..
CauYps4    .GYDTSEGCALGVIDGG.....NKSAILGDVFLRSAYAVFDFDNYEIALAQAAGEEKQQK
CaSap9     ....SKSTCILGVMQQSSS...SSYMLFGDNILRSAYIVYDLDDYEVSLAQVSYT..NKE
CauYps1    .....RSYCFLGIMPQASSNRGTSYAVLGGNFLRSAYVVYDLDDYTISLAQANHS..NEE
CaSap10    G......QCYLAIMSNSVIGG..GGILFGDDILRQIYLVYDLQDMTISVAPVVYT..EDE
ScYps1     GTT.....CLLGIIPTSDDT....GTILGDSFLTNAYVVYDLENLEISMAQARYN.TTSE
CgYps1     GDS.....CILGIMPQSGG.....GIILGDSFLNSAYVVYDLENYEISMAQANYA.GGQE
ScYps2     SRSN...ICILGIAPQSDP.....TIILGDNFLANTYVVYDLDNMEISMAQANFS.DDGE
ScYps3     VGT.....CVLAIIPQAGNA....TAILGDSFLRNAYVVYDLDNYEISLAQAKYG.TGKE
ScYps6     KDR.....CVLNVK.QSES.....TYMLGDAFLVDAYVVYDLENYEISIAQASFN.NQEE
CgYps3     KRKG...KCYLEIRYHEELSK..TGMLLGDIFMRHVYSVFNMEDMEVSFAVANGNDSAPL
CgYps10    KKSG...KCYLDLMVAPPNNG..PGLLLGDTFLRQVYTVFDLEAQELSIARANFNSSLPA
CgYps9     KHKG...KCYLQFSALDGIN....FALLGDNFMNNVYTVFNLDDKELSLAQANYNSSLKP
CgYps6     KVKG...KCYLEIIPSGAES...DLVILGDNFMRSVYTVFDLEANEVSIAQANFNSSLPA
CgYps8     KHKG...KCYLQFAVHEDTSN..DAIIFGDNFLRSVYLVYNLEDKEISIAPANFNSSLPE
CgYps4     KIKG...KCYLQMQARDDIE....FAALGDIFLRHVYVVYNLEDEEISIANANFN.YSTP
CgYps5     KHKG...KCYLPCVS.QESS....FFLLGDSFMRHVYTVFDLDDKEISIAQANFN.STKE
CgYps2     KSDDD..NCYVTFMPQGEPG.....ILLGDNFLRNAYIVYDLEDMEISIAQANFN.GGAE
CgYps11    KIKG...KCYINVAPTGSDF.....AILGDDVIRYAYMVFDLEDREISLAQASFNDTLPE
ScYps7     SSGQE..ACFLTLYANTNTG....VNILGEAFIKNIYMAMDLEDNTIAIAQAKKVEDD..
CgYps7     NTGQE..ACVLQLYPNTFTG....YNVLGQAFMKNAYLAVDLEGKGVAMGQAANFKSGER
CauYps6    QG.....YCASKLQGTSEST....SWILGASFLNQFYTIFDLQNARVGFAPRVDS.....
CauYps7    FSGEAK.ACILNVSPSSYLG....YTSLGLPFLTNAYFAVDNDGKHIALGNHNKNYTANT
```

C4

```
                                      β26
CtSap1                                -->

CtSap1     ............................DISAL...........................
CaSap5     ............................DIVAIN..........................
CauYps5    ...........................VNIEEISNAIPSAVAAEGFNDTSVDYYLNYTG.
CauYps3    ...........................ADIEEVTGDIPGASKAQYYDFTSVSEQFQYTKS
CauYps2    ...........................PDIEEIVEDIPRAINADEFNETEIVNSMVWISD
CauYps4    E.........................GSHIVTISSGIPSASPAPEYSSTTLSDVLLYNTT
CaSap9     ............................SIEVIG...........ASGITNSSGSGTTSS
CauYps1    ............................DIEIIQNSIPDAVSALGASATPSFGGSSEPES
CaSap10    ............................DIEEILNPNEDQNEVPTSTSFTQSASSSGSQP
ScYps1     ............................NIEIITSSVPSAVKAPGYTNTWS.TSAS.IVT
CgYps1     ............................DIEVISSSVPGAVRAPGFSSTWS.TLATSFNT
ScYps2     ............................YIEIIESAVPSALKAPGYSSTWS.TYES.IVS
ScYps3     ............................NVEVIKSTVPSAIRAPSYNNTWS.NYAS.ATS
ScYps6     ............................DIEVISDTVPGATPAPGYFSTWV.YKPGSPIG
CgYps3     ............................QIEAIISDVPSAVKAPQYYNTFA.SSNLPSTV
CgYps10    ............................DVEEIKSTVPSAIKAPQYYNTFS.AYDNATQV
CgYps9     ............................DIEEIKDTVPSAVLAPQYYNTFS.ADPTATAV
CgYps6     ............................QIEEIKSTVPSAVKAPQYYNTFS.AIASISSV
CgYps8     ............................NIEPIKSTVPSAIRAPQYYNTYS.EHATATAV
CgYps4     ............................DIQEIVSTVPGAVKAPQYYNTYS.VYPTATAV
CgYps5     ............................DIEEISSTVPSAVKAPQYYSSYV.DFPTEISV
CgYps2     ............................NIEAIVKNVPSAVKAPGYSASFT.EFPSSYAT
CgYps11    ............................DIEVVVSTIPGATRASAYSETYSPTFAVPSAS
ScYps7     ...........AVTEETNET...TASTIIKKIKSGYIPYAKVMNSSNTRNLTLYPSYRSG
CgYps7     KEIITRQASIVTFTDSLNKTSVATFSTVPSSMTSGYIPYATYESDSAYDSMQLYPSKVGS
CauYps6    .............................FQILSANTSGNSTSSSSSSTSTSNTSSGAPS
CauYps7    ............................DFSLQSGSSKSNKSDDGASLIASGTIPFASTY
```

```
CtSap1

CtSap1     ............................................................
CaSap5     ............................................................
CauYps5    ..........................TSWETFAADDASITSLFS................
CauYps3    GSKSKLVVFTESFSDPVPEATPLTTDDSWSAESNSDFDYSSIISATGDLPEGLNSYPGLG
CauYps2    RPGY............TVQGGLGSSTTSVSTSSGEDTPESTITSAT..............
CauYps4    GSSYYYS..........TTTFGNHVTVAAESTSANGTNNTHAYPSY..............
CaSap9     SGTSTST............................STRHSAGS.................
CauYps1    DSSVTAV............................STSNVKNS.................
CaSap10    SSTISGE............................NMDKNTTSSSSGNC...........
ScYps1     GGNIFTV................NSSQTAS............FSGN............LT
CgYps1     QGDIFTV................QAAATVSGSATNTGTGRATATGNS.....NSTRSTTS
ScYps2     GGNMFST................AANSSISYFASTSHSATSSSSSKGQKTQTSTTALSIS
ScYps3     GGNIFTT................VRTFNGTSTATTTRSTTTKKTNS............TT
ScYps6     TGDFINV................SWTSYSEFSQYKSLLATAAQSDD............AS
CgYps3     TNDIFAS............................TATASMEPPSSVL........ESLK
CgYps10    VGNIFDA............................TATASMAPKN..............Q
CgYps9     TGNIFAP............................EATMSMAAPAN............AS
CgYps6     TGDIFGP............................EATAYMAPPAN.............V
CgYps8     TGDIFAP............................EATMSMAPIN..............K
CgYps4     TGDVFTP............................EATMSMSN.................
CgYps5     TGDIFAP............................QATEFVPKPNN.............T
CgYps2     TGNIFSG...........................YNSTNSTGSAG............SKT
CgYps11    SSGNTSS............................NGSTNLTRRFE..............
ScYps7     YMFTVPG................QLT.AAYSNGVITGAGRSFYDTSRASTSARPSSTQFD
CgYps7     RITSIPG................QIAGTVYPGGVIAGDGRSFYDTSRTTTKLQTS.TQFD
CauYps6    NQSTSPP.................................KQHSG...............
CauYps7    NYTSSATLTIN............PANSSAAEAVLTKYSLASIVSGEVVVSGHSSSTVGPL
```

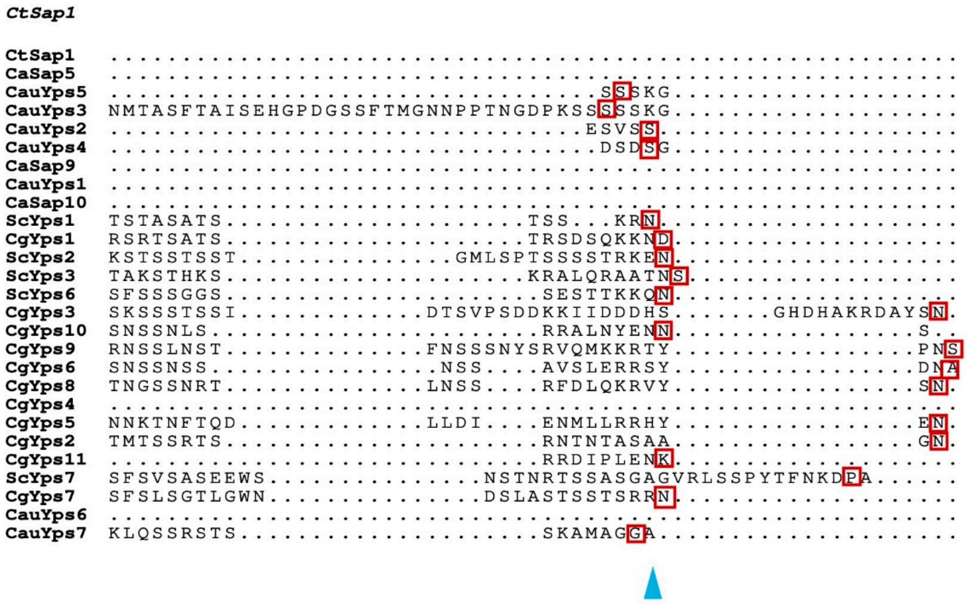

Figure S2. Predicted secondary structure and motifs of yeast yapsins. Arrows: β-sheet, spiral: α-helix, red arrowhead: monobasic or dibasic residues of Kex processing or autoprocessing-sites, blue arrow head: dibasic or monobasic residues implied of autoprocessing involved in the sheddase activity of yapsins, D1 and D2: catalytic aspartic residues; C1-2 and C3-4: Cysteines involved in disulfide bridges, dark and light pink lines: removed internal loop, red squared: ω: omega site of GPI anchorage. SAPs of *C. tropicalis* (CtSap1, 1J71) and *C. albicans* (CaSap5, 2QZX) were the templates. *S. cerevisiae* (Sc), *C. glabrata* (Cg) and *C. auris* (Cau). The analysis was performed with the ENDscript v3.0 server.

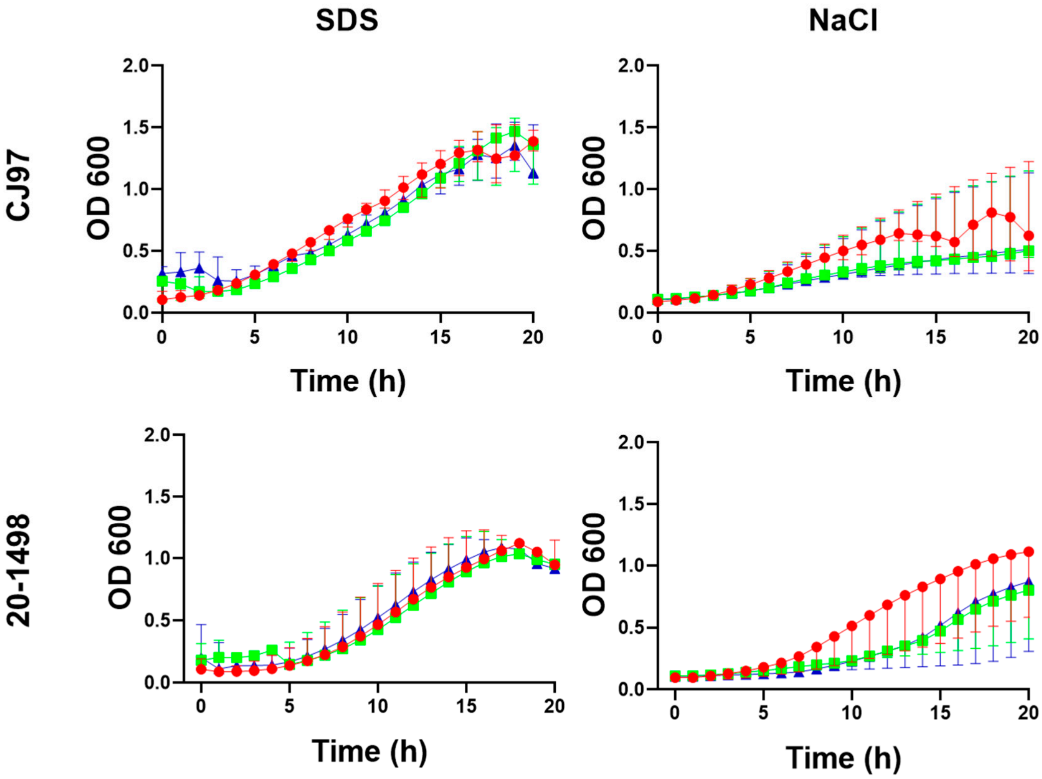

Figure S3. Effect of pepstatin A on the growth of *C. auris* in the presence of different compounds. Yeasts were grown in YPD medium at 37°C with and without 0.05% SDS or 1.5 M NaCl, supplemented or no with 25 µM pepstatin A, red and blue curves, respectively. Green: control with 0.01 % methanol used as the vehicle for pepstatin A. At least three replicates were performed. Bars indicate standard deviation.

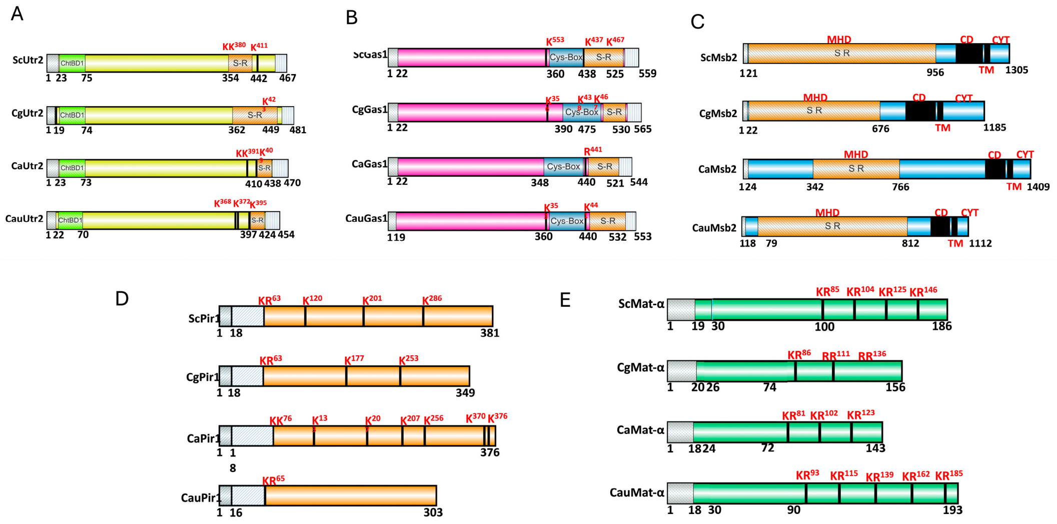

Figure S4. Predicted protein targets of Yps1 of *C. auris*. Homologous targets of Yps1 of *S. cerevisiae* and Sap9 of *C. albicans* were identified, and those of *C. auris* and *C. glabrata,* and they present probable monobasic or dibasic sites of recognition by these proteases. **A)** The GPI-anchored chitin transglycosidases Utr2 (Crh2) carries at least one putative cleavage site of Yps1 and Yps2, involved in the release of the catalytic domain (green rectangle). ScUtr2 (AJV34305.1), CgUtr2 (XP_445272.1), CaUtr2 (XP_721748.1), CauUtr2 (XP_028889825). **B)** The GPI-anchored β-1,3-glucanosyltransferase Gas1 of *C. auris* shows probable monobasic recognition sites by Yps1, conserved with those of the ScGas1, one of each side of the Cys-box (blue rectangle), a carbohydrate binding module required for Gas1's role in cross-linking cell wall glucans [43,66,67,70]. ScGas1 (AJS80525.1), CgGas1 (XP_449946.1), CaGas1 (XP_719043.1), CauGas1 (XP_028892777). **C)** The transmembrane sensor of stress Msb2 contains, although not well conserved in the ScMsb2, probable dibasic residues in the CD domain (cleavage domain, black rectangle), probably recognized by Yps1. In *S. cerevisiae,* this process releases the inhibitory domain (HMD, orange rectangle), thereby activating the MAK pathway [66]. ScMsb2 (NP_011528), CgMsb2 (XP_446100), CaMsb2 (XP_722538), CauMsb2 (XP_028892564). **D)** In contrast to other yeasts, the cell wall protein Pir1 of *C. auris* showed only one cleavage site by Yps1, probably involved in processing the

prosegment (grey rectangle), the internal repeats (orange region) [4, 46,70.] which seems to link to the β-1,3-glucans could be not cleavage by CauYPs. ScPir1 (ONH77051.1), CgPir1 (XP_447520), CaPir1 (XP_712603), CauPir1 (XP_054558182). **E)** The pro-α-mating factor (proMatα) is matured to the secreted Matα of 13 amino acids, after processing by Ste13 and the Kex2 serine carboxypeptidase, which recognizes the X-Ala and the dibasic sites respectively. In the absence of Kex2, Yps1 mature proMatα at the dibasic residues [5, 46, 47, 68]. Although, it is not sexual reproduction until now, *C. auris* of clades II and III carries *MTLα* locus, while *C. auris* of clades I and IV carries *MTLa* locus, and proMatα might be processed similarly to that of *S. cerevisiae*. ScMatα (AGW24899.1), CgMatα (XP_446929.1), CaMatα (KAL1575323.1), Cau Matα (QRG37869.1; clade V). ScMsb2 (NP_011528), CgMsb2 (XP_446100), CaMsb2 (XP_722538), CauMsb2 (XP_028892564). N-terminal gray rectangle: signal peptide, Sc: *S. cerevisiae*, Cg: *C. glabrata*, Ca: *C. albicans*, Cau: *C. auris*. IBS v2 was used to draw images.

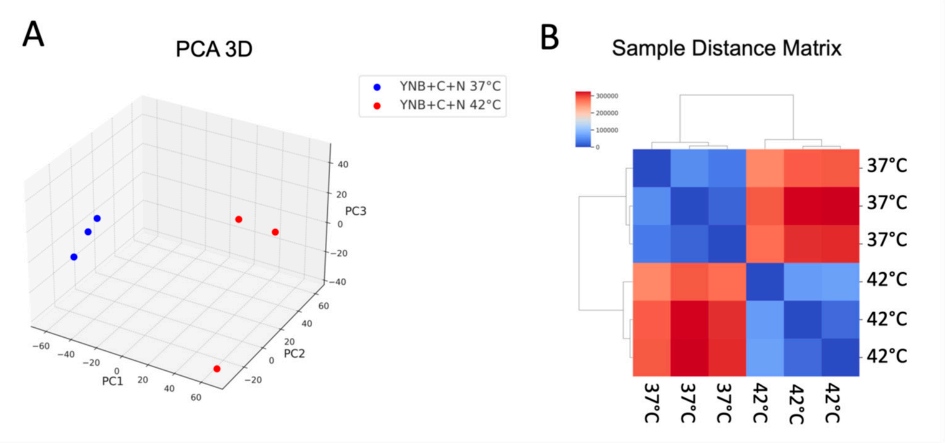

Figure S5. Global transcriptomic analysis of *C. auris* 20-1498 (clade IV). Yeasts were cultured in YNB+C+N medium at 37°C and 42°C for 6 h. **A)** Three-dimensional principal component analysis (PCA 3D) based on normalized log2 counts. The 37°C condition is shown in blue and the 42°C condition in red. **B)** Sample distance matrix represented as a hierarchical heatmap. The color scale indicates the degree of dissimilarity: blue reflects differences between samples, while red indicates similarities.

68. Julius, D.; Schekman, R.; Thorner, J. **1984**. Glycosylation and processing of prepro-α-factor through the yeast secretory pathway. *Cell*, **36**, 309–318. https://doi.org/10.1016/0092-8674(84)90224-1

70. Ribeiro, R. A.; Bourbon-Melo, N.; Sá-Correia, I. **2022**. The cell wall and the response and tolerance to stresses of biotechnological relevance in yeasts. *Front Microbiol*, **13**, 953479. https://doi.org/10.3389/fmicb.2022.953479

Table S1. Accession numbers and data generated from the transcriptomic analysis of *C. auris* at 42°C

**Gene Name**	**Gene ID**	**log2FoldChange**	**padj**	**Accession number in *C. albicans***
WSC2	CJJ07_005215	-0.6820372	3.1495E-31	C6_02670C_A
FKS1	CJJ07_005267	-0.8426184	9.796E-73	C1_02420C_A
FKS2	CJJ07_001502	-1.1000412	5.6872E-86	CR_00850C_A
SAC7	CJJ07_001232	-0.6068461	3.167E-21	C7_00080C_A
RLM1	CJJ07_000609	-0.8143157	4.169E-13	C4_01260W_A
GSC1	CJJ07_005267	-0.8426184	9.796E-73	C1_02420C_A
KRE1	CJJ07_002988	-1.5080619	1.424E-115	CR_03790C_A
KRE6	CJJ07_003483	-2.8166711	1.362E-230	C3_05830W_A
SKN1	CJJ07_003482	-0.9456505	8.7189E-50	C3_05810C_A
CHS1	CJJ07_001822	-0.6406752	1.4534E-16	C7_02770W_A
CHS2	CJJ07_000439	-0.9595887	1.4998E-60	CR_09020C_A
CHS3	CJJ07_001556	-0.8905505	3.045E-98	C1_13110C_A
MNN2	CJJ07_001439	-1.0812497	1.8919E-98	C1_10720C_A
DPM1	CJJ07_004165	-0.8937436	1.0814E-13	C1_08010W_A
ERG10	CJJ07_002606	-1.7665148	1.18E-254	C2_04310W_A
ERG1	CJJ07_001622	-1.5358262	6.944E-148	C1_08590C_A
ERG6	CJJ07_003408	-0.8918201	5.7339E-72	C3_02150C_A
ERG13	CJJ07_002854	-0.9715977	1.7034E-50	CR_09160C_A
ERG26	CJJ07_000643	-0.8207397	1.9795E-41	C4_06270C_A
ERG2	CJJ07_003239	-0.8083305	2.2444E-39	C1_00800C_A
ERG7	CJJ07_005369	-0.6898544	3.1379E-11	C2_02460W_A
ATG17	CJJ07_000949	0.63848537	0.00107927	C1_02910C_A
ATG2	CJJ07_001128	-0.9564087	4.3373E-53	C2_06010W_A
ATG13	CJJ07_000638	-0.7484017	8.258E-18	CR_02910W_A
ATG10	CJJ07_003421	0.84122876	1.9589E-06	C3_02930W_A
ATG11	CJJ07_003172	0.94317433	1.7679E-22	C4_01790W_A
TSC11	CJJ07_002277	0.59669969	1.9069E-12	CR_07580C_A
SIP2	CJJ07_005601	-0.7793599	1.5344E-11	C2_09230C_A
NPR3	CJJ07_001862	0.58817014	9.0134E-08	CR_08680C_A
ELM1	CJJ07_000969	0.80043982	2.6969E-10	C3_00790W_A
GCN2	CJJ07_003821	0.65355325	4.8963E-14	C7_01330C_A
TAP42	CJJ07_001882	0.71932847	6.3168E-20	C4_01670C_A
TIP41	CJJ07_003283	-0.5876229	8.5218E-07	C5_04520W_A
UME6	CJJ07_003670	-1.2489014	2.6666E-18	C1_06280C_A
CCZ1	CJJ07_000807	-0.6946554	8.5656E-08	C6_03580W_A
VPS16	CJJ07_001073	0.87541873	3.4313E-16	C1_04540C_A
VPS41	CJJ07_000118	0.72345176	1.185E-17	C1_09920W_A

AVT6	CJJ07_001046	0.63561435	4.4974E-16	C6_04110W_A
PEP4	CJJ07_003537	-0.6862827	2.0794E-25	C2_07400C_A
PRB1	CJJ07_001437	-0.6768821	1.1791E-52	C7_03860W_A
PRC1	CJJ07_002829	-0.6431247	5.9868E-43	C7_03360W_A
SFK1	CJJ07_000791	1.16299958	1.27E-12	C4_02720C_A
YPD1	CJJ07_005152	1.08453095	1.0074E-68	C1_07240W_A
HOG1	CJJ07_004739	-0.7046391	9.208E-17	C2_03330C_A
YDJ1	CJJ07_000538	1.04613334	4.8196E-97	CR_04200W_A
HSP60	CJJ07_002013	0.6064541	4.3417E-39	CR_06490C_A
CPR6	CJJ07_002261	0.80259524	1.3326E-07	CR_10670W_A
HCH1	CJJ07_002547	0.60157556	3.2314E-15	C6_01860C_A
SGA1	CJJ07_001987	0.60794569	7.7964E-14	C3_01320C_A
ENG1	CJJ07_002251	0.63741216	7.6934E-34	C1_03680W_A
FAT1	CJJ07_002356	0.69027402	3.0289E-13	C6_00740W_A
HSP104	CJJ07_002319	1.1271102	5.541E-250	CR_08250C_A
SSA1	CJJ07_004645	1.00960566	2.371E-162	C1_13480W_A
HSF1	CJJ07_003569	1.13497058	4.8956E-88	C1_09170W_A
UTR2	CJJ07_003210	-1.029056	8.149E-126	C3_01730C_A
MSB2	CJJ07_001204	-0.7124971	5.5436E-16	C2_01780W_A
YWP1	CJJ07_003597	-2.0149375	1.061E-31	C2_08590W_A
RBT5	CJJ07_000323	-1.8945204	9.5915E-34	C4_00130W_A
ECM33	CJJ07_003041	-0.6048541	4.1985E-55	C1_03190C_A
CEK1	CJJ07_002301	-0.77857	7.1994E-09	C4_06480C_A
HGT1	CJJ07_005266	0.97331208	1.1643E-62	C1_01980W_A
RAG1	CJJ07_003380	-1.5701959	0.000001	CR_03450W_A
HXT10	CJJ07_004946	0.58632524	7.5296E-15	C1_02110C_A
PCK1	CJJ07_002193	1.10616362	1.374E-106	CR_00200W_A
PGA7	CJJ07_000324	-1.7143595	5.702E-118	C4_00120W_A
FRP1	CJJ07_000326	-0.735235	1.2274E-39	C4_00110C_A
AOX2	CJJ07_005061	1.36334829	8.3E-229	C1_09150W_A
